# *Clostridioides difficile* binary toxin CDT induces biofilm-like persisting microcolonies

**DOI:** 10.1080/19490976.2024.2444411

**Published:** 2024-12-24

**Authors:** Jazmin Meza-Torres, Jean-Yves Tinevez, Aline Crouzols, Héloïse Mary, Minhee Kim, Lise Hunault, Susan Chamorro-Rodriguez, Emilie Lejal, Pamela Altamirano-Silva, Déborah Groussard, Samy Gobaa, Johann Peltier, Benoit Chassaing, Bruno Dupuy

**Affiliations:** a Pathogenesis of Bacterial Anaerobes, Department of Microbiology, Institut Pasteur, Université Paris-Cité, UMR-CNRS 6047, Paris, France; b Image Analysis Hub, Department of Cell Biology and Infection, Institut Pasteur, Université Paris Cité, Paris, France; c Biomaterials and Microfluidics Core Facility, Department of Developmental and Stem Cell Biology, Institut Pasteur, Université Paris Cité, Paris, France; d Antibodies in Therapy and Pathology, Department of Immunology, Institut Pasteur, Paris, France; e Institute for Integrative Biology of the Cell (I2BC), Université Paris-Saclay, CEA, CNRS, Gif-sur-Yvette, France; f Centro de Investigación en Enfermedades Tropicales, Universidad de Costa Rica, San José, Costa Rica; g Animalerie Centrale, Institut Pasteur, Paris, France; h Microbiome-Host Interactions, Department of Microbiology, Institut Pasteur, Université Paris Cité, INSERM U1306, Paris, France; i Mucosal Microbiota in Chronic Inflammatory Diseases, INSERM U1016, CNRS UMR 8104, Université Paris Cité, Paris, France

**Keywords:** CDT binary toxin, *Clostridioides difficile* Infection (CDI), relapse, biofilm, gut persistence, mucin-associated microcolonies, antibiotic resistance

## Abstract

Clinical symptoms of *Clostridioides difficile* infection (CDI) range from diarrhea to pseudomembranous colitis. A major challenge in managing CDI is the high rate of relapse. Several studies correlate the production of CDT binary toxin by clinical strains of *C. difficile* with higher relapse rates. Although the mechanism of action of CDT on host cells is known, its exact contribution to CDI is still unclear. To understand the physiological role of CDT during CDI, we established two hypoxic relevant intestinal models, Transwell and Microfluidic Intestine-on-Chip systems. Both were challenged with the epidemic strain UK1 CDT^+^ and its isogenic CDT^−^ mutant. We report that CDT induces mucin-associated microcolonies that increase *C. difficile* colonization and display biofilm-like properties by enhancing *C. difficile* resistance to vancomycin. Importantly, biofilm-like microcolonies were also observed in the cecum and colon of infected mice. Hence, our study shows that CDT induces biofilm-like microcolonies, increasing *C. difficile* persistence and risk of relapse.

## Graphical abstract

**Figure:**
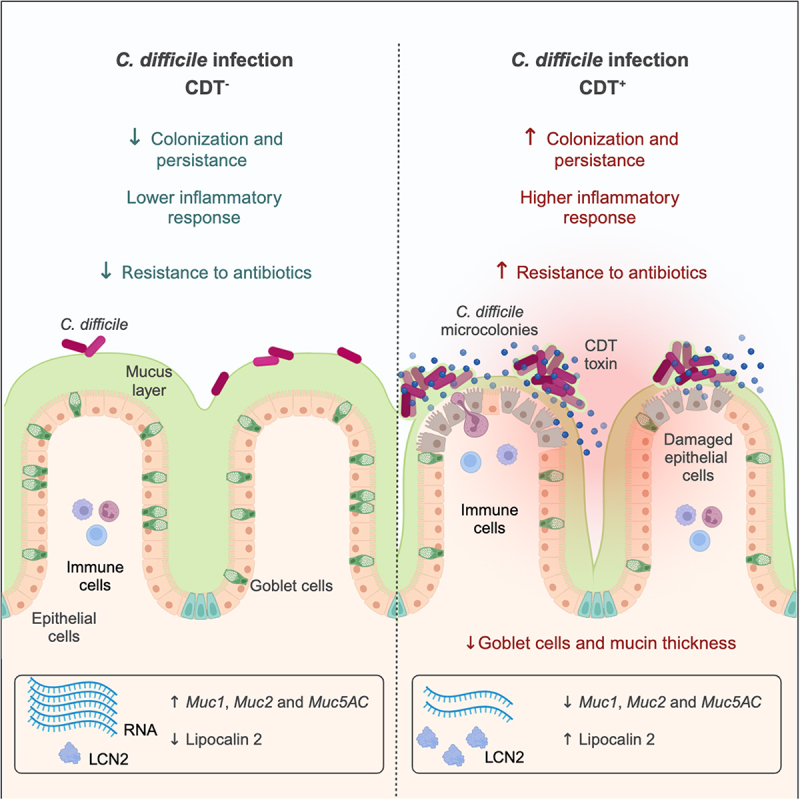
***Clostridioides difficile* CDT binary toxin induces the formation of mucin-associated microcolonies with biofilm properties.** These microcolonies increase *C. difficile* colonization and persistence in mice. In addition, mucin increases CDT levels and biofilm formation. CDT, in turn, induces transcriptional changes of mucin-associated genes resulting in a reduction of mucin thickness and goblet cells in the colon.

## Introduction

*Clostridioides difficile* is an obligate anaerobic spore forming bacterium and the leading cause of antibiotic-associated diarrhea.^1^ Antibiotic treatments alter the gut microbiota and allow germination of *C. difficile* spores present in the gut.^2,3^ A major challenge in managing CDI is the high rate of relapse.^4–6^ Recurrent CDI (rCDI) due to relapse or reinfection occur in 20–35% of the cases in the 2 months following the initial episode.^7–9^ After a first relapse episode, patients have a higher risk (around 60%) of presenting a second relapse.^7,8,10^ Recent findings indicated that spores are able to enter into epithelial cells and contribute to *C. difficile* persistence and rCDI.^11^ However, inhibition of spore entry into epithelial cells only delayed relapse,^11^ indicating that other mechanisms are involved in rCDI.

The key virulence factors of *C. difficile* involved in the host intestinal damages are the two toxins TcdA and TcdB. Both toxins modify and inactivate Rho and Rac GTPases,^12^ triggering the disruption of the cytoskeleton, the breakdown of tight junctions and the subsequent loss of epithelial integrity.^13^ In addition, 17–23% of the clinical strains produce a third toxin, namely the *C. difficile* transferase toxin (CDT) or binary toxin. This toxin is composed of two separate toxin components: CDTa, the enzymatic ADP-ribosyl-transferase that depolymerizes F-actin, and CDTb, the cellular binding component that forms heptamers after proteolytic activation and translocates CDTa into the cytosol. The ADP ribosylation of actin by CDTa results in the formation of long microtubules protrusions that form a tentacle-like network on the surface of epithelial cells.^14^ The actin depolymerization also leads to a misguided secretion of vesicles containing extracellular matrix (ECM) proteins. Together, the microtubule protrusions and ECM-containing vesicles increase the adherence of *C. difficile* to epithelial cells.^14,15^

Despite advances in understanding how CDT affects host cells, its role in infection and disease remains unclear.^16^ To date, CDT has been shown to enhance colonization^17^ and CDT^+^ strains correlate with an increased virulence leading to more severe diarrhea, increased pain, higher fatality rates, and higher rCDI.^18–24^ As studies addressing these questions suffered some limitations or bias, such as using (i) insertional CDT gene mutants with possible polar effects,^24^ (ii) originally non-toxigenic TcdA^−^TcdB^−^CDT^+^ strains,^17^ (iii) non-isogenic strains,^25^ or (iv) a short infection time (2–3 days).^26^

In this study, we used *C. difficile* epidemic strain UK1 and generated *tcdA*^−^*tcdB*^−^*cdtAB*^±^ isogenic mutant strains to elucidate the role of CDT during CDI. We show that *C. difficile* binary toxin CDT has a role in colonization through the formation of 3D biofilm-like microcolonies structures. These microcolony structures have biofilm-like properties such as increased resistance to antibiotics. Our results support the implication of CDT in *C. difficile* long-term colonization and suggest that the 3D biofilm-like CDT-dependent structures are involved in *C. difficile* persistence in the gut. These findings provide evidence that CDT could play a crucial role in *C. difficile* relapses.

## Materials & methods

### Bacterial strains and culture conditions

Bacterial strains and plasmids used in this study are listed in Table S1. *Clostridioides difficile* strains were routinely cultured on BHI agar (Difco), BHI broth (Difco), or TY broth (Bacto tryptone 30 g.L^−1^, yeast extract 20 g.L^−1^, pH 7.4) at 37°C in an anaerobic environment (90% [vol/vol] N_2_, 5% [vol/vol] CO_2_, and 5% [vol/vol] H_2_). When necessary, *C. difficile* culture media were supplemented with cefoxitin (Cfx: 25 mg/liter), cycloserine (Ccs: 250 mg/liter), thiamphenicol (Tm: 7.5 mg/liter), and erythromycin (Erm: 5 mg/liter). *Escherichia coli* strains were cultured at 37°C in LB broth or LB agar (MP Biomedicals), containing when needed chloramphenicol (Cm: 25 mg/liter) and ampicillin (Amp: 100 mg/liter).

### Construction of *C.* *difficile* mutant strains

All primers used in this study are listed in Table S2. A pathogenicity locus (PaLoc)-deleted strains of *C. difficile* 630∆*erm* and UK1 that lacked the *tcdB*, *tcdE*, and *tcdA* genes were generated.^27^ A CDT locus (CdtLoc)-deleted strain UK1 strain, lacking the *cdtA* and *cdtB* genes (designed as CDT^−^), was then generated in the Δ*toxAB* background. The deletion mutants were created using a toxin-mediated allele exchange method.^28^ Briefly, approximately 850 bp of DNA flanking the region to be deleted were amplified by PCR from *C. difficile* UK1 and 630∆*erm*. Purified PCR products were cloned into the PmeI site of the pMSR0 vector using NEBuilder HiFi DNA Assembly (New England Biolabs). The resulting plasmid was transformed into *E. coli* strain NEB10β (New England Biolabs) and insert verified by sequencing. Plasmids were then transformed into *E. coli* HB101(RP4) and transferred by conjugation into the appropriate C. *difficile* strains. Transconjugants were selected on BHI supplemented with cycloserine, cefoxitin, and thiamphenicol. Allelic exchange was performed as described previously.^28^

### Cell culture

Caco-2 cells (clone TC-7) and HT29-MTX cells were provided by Nathalie Sauvonnet from Institut Pasteur, Paris, France. Cells were grown in Advanced Dulbecco’s Modified Eagle Medium (ADMEM, Gibco) supplemented with 10% FBS (fetal bovine serum, Biowest) and L-glutamine (Gibco) in 5% CO_2_ at 37°C. Cells were kept in culture up to passage number 15.

### Germ-free mice experiments

C57/BL6 6-week-old gnotobiotic male and female mice from Institut Pasteur Animal facilities were used. They were housed in the Institut Pasteur animal facilities accredited by the French Ministry of Agriculture to perform experiments on live rodents. Mice were acclimated on independent isolators (one isolator per strain) for a week prior to *C. difficile* challenge. Later mice were challenged with *C. difficile* spores (2 × 10^3^ per mice) by oral gavage. Mice health was monitored daily as described previously.^29^ Progression of disease was assessed via Body Condition Scoring and body mass measurements.^30^ Mice were followed to a 13 day post *C. difficile* challenge.

### Transwell intestinal model (TIM)

Caco-2 cells or Caco-2-HT29-MTX cells co-culture were seeded into 12-well Transwell inserts (pore size 0.4 µm, Corning) at a density of 2 × 10^5^ cells/cm^2^ and cultured for 18 days at 5% CO_2_ at 37°C. Cell culture media was changed three times a week.

### Intestine-on-a-chip model (IoC)

IoC-associated instrumentation and software were obtained from Emulate (Human Emulation System, Boston MA). Chips were prepared following manufacturer’s instructions and as described previously.^31,32^ Briefly, chips were activated using ER1/ER2 solution (Emulate, 0.5 mg/mL) under UV for 20 min (36 W, 365 nm) then washed once with ER2 solution (Emulate), followed by 2 PBS washes (Gibco). Chips were coated overnight (ON) at 4°C with ECM composed of 200 ug/mL of human Collagen IV (Sigma) + 100 ug/mL of Matrigel (Corning). ECM materials were washed twice with PBS followed by cell culture media. Caco-2 cells were added to the epithelial channel at a concentration of 10^6^ cells/mL density. Caco-2 and HT29-MTX cells co-culture was prepared by adding 8 × 10^5^ cells/mL of Caco-2 + 2 × 10^5^ cells/mL of HT29-MTX (4:1 ratio). Cells were incubated for 1 day under static conditions at 37°C with 5% CO_2_. After the cells adhere to the substrate, chips were gently washed with warm cell culture media to remove non-attached cells, then connected to the primed Pods (Emulate). Pods-chips were kept in the Zoë (Emulate) with a flow of 30 uL/h on the top and bottom channels for 1 day, then adding a stretch (10%, 0.15 hz) for 6 days. Cell culture medium reservoirs were refilled every 3 days.

### Lactate dehydrogenase release assays

To measure Lactate dehydrogenase release from Caco-2 cells or Caco-2 co-cultured with HT29-MTX cells in the TIM or IoC models, we used the commercial kit CytoTox 96 Non-Radioactive Cytotoxicity Assay (Promega) according to manufacturer’s instructions. The relative cytotoxicity obtained from cultures under normoxia conditions (at 5% CO_2_) was considered as 0% and these values were compared to hypoxia conditions over time (4% O_2_ and 5% CO_2_).

### TIM infection under hypoxia

*C. difficile* strains were cultured ON on TY broth, the next day ON cultures were diluted (1:50) with new fresh media to obtain exponential phase bacteria (*λ*_600 nm_ 0.3 to 0.5). Bacteria were diluted to 10^6^ bacteria/mL in equilibrated ADMEM before infection. Cells were equilibrated 1 h before infection under hypoxia conditions (4% O_2_, 5% CO_2_), then wells were infected with 500 uL of bacterial suspension (10^6^ bacteria/mL).

### TIM adhesion assays

Infection conditions were kept as indicated previously for TIM infection under hypoxia conditions (4% O_2_, 5% CO_2_). After 3, 6, 18 or 24 h of incubation cells were washed three times with 500uL of PBS (Gibco) to eliminate non-adherent bacteria. Cells and adherent bacteria were diluted in 500 uL of PBS (Gibco) and recovered by scraping the Transwell wells with 1 mL tips and centrifugation (5 min at 5 000 rpm). Adherent bacteria were serially diluted and plated on TY agar plates, incubated for 48 h at 37°C under anaerobic conditions.

### IoC infection under hypoxia

*C. difficile* strains were cultured ON on TY broth, the next day ON cultures were diluted (1:50) with new fresh media to obtain exponential phase bacteria (*λ*_600 nm_ 0.3 to 0.5). Bacteria were diluted to 10^6^ bacteria/mL in equilibrated ADMEM before infection. IoC was equilibrated 6 h before infection by decreasing O_2_ levels each hour (18%, 15%, 12%, 9%, 6%, 4%; with 5% CO_2_) in the housing cell culture incubator. IoC chips were disconnected from the pods and infected under static conditions (no flow, no stretch) with 50 uL of bacterial suspension (10^6^ bacteria/mL). After 1 h 30 min, chips were reconnected to the Pods and reintroduced in the Zoë with a flow of 30 μL/h (no stretch during infection).

### TIM and IoC immunostaining

TIM and IoC were fixed with 4% of paraformaldehyde (Electron Microscopy Sciences) diluted in PBS with Ca^2+^ and Mg^2+^ (Gibco) for 30 min. After fixation, Transwell and chips were washed three times with PBS and stored at 4°C. For the IoC transversal sections, the chips were cut in 300-µm-thick slices using a vibrating blade microtome (VT1000S, Leica). IoC sections and Transwell were permeabilized with 0.1% Triton X-100 in PBS with Ca^2+^ and Mg^2+^ (Gibco) for 20 min at room temperature (RT) and then washed three times with PBS. Later, blocking solution (2% BSA in PBS with Ca^2+^ and Mg^2+^) was added for 1 h at RT.

### Production of lmw‐SlpA specific monoclonal antibodies NF10 and QD8

Knock-in mice expressing human antibody variable genes for the heavy (VH) and kappa light chain (Vκ) were previously described^33,34^ and provided by Regeneron Pharmaceuticals to be bred at Institut Pasteur. BALB/c mice were purchased from Janvier Labs. All animal care and experimental procedures were conducted in compliance with national guidelines. The study, registered under #210111, was approved by the Animal Ethics Committee of CETEA (Institut Pasteur, Paris, France) and by the French Ministry of Research. BALB/c and VelocImmune mice were injected intraperitoneally on days 0, 21, and 42; with 50 μg of either recombinant LMW630 mixed with 200 ng/mouse pertussis toxin (Sigma-Aldrich, MO, USA) for NF10 production or with 50 μg of each of five recombinant LMWs in alum mixed with 200 ng/mouse pertussis toxin (Sigma-Aldrich, MO, USA) for QD8 production. An enzyme-linked immunosorbent assay (ELISA), previously described,^35^ was performed to measure serum responses to antigens and the three immunized animals with the highest serum titers were boosted with the same preparation. Four days later, splenocytes were fused with myeloma cells P3X63Ag8 (ATCC, France) using a ClonaCell-HY Hybridoma Kit, according to the manufacturer’s instructions (StemCell Technologies, Canada). Culture supernatants were screened using ELISA,^35^ and antigen-reactive clones were expanded in serum IgG-free RPMI-1640 (Sigma-Aldrich) into roller bottles at 37°C. After 14 days, the supernatants were harvested by centrifugation at 2 500 rpm for 30 min and filtered through a 0.2 μm filter. Antibodies were purified by Protein A affinity chromatography (AKTA, Cytiva, Germany), as described previously.^36^

### Spinning disk fluorescence microscopy

Images were performed in a Nikon Ti-E inverted microscope equipped with a Perfect Focus System (TI-ND6-PFS Perfect Focus Unit) and a Yokogawa confocal spinning disk unit (CSU-W1) using a 60X/1.42 NA oil objective. A Z-stack of 300 to 800 planes with 0.3 µm z-steps was acquired sequentially in four channels (Da/Fi/Tr/Cy5-4×-B, Finkel Quad FF01–440/521/607/700).

### Minimal inhibitory concentration (MIC) determination and antibiotic resistance assays

MICs were determined by broth microdilution as described before.^37^ Briefly, a 96-well plate containing twofold dilutions of desired antibiotic were inoculated with ON culture diluted to a final *λ*_600 nm_ of 0.05 in ADMEM (Gibco) supplemented with 10% FBS (Biowest) and L-glutamine (Gibco). After 24 h at 37°C, MIC was determined by measuring λ_600 nm_ in a plate reader (Promega GloMax Explorer). Supplemented ADMEM medium was used as a blank.

For the antibiotic resistance assays in TIM, infections were performed as indicated previously and 24 h p.i, antibiotics were added at 1×, 10×, and 100× the MIC. MIC for fidaxomicin was defined as 1 μg/mL and for vancomycin as 12.5 μg/mL. After 24 h of antibiotics treatment, resistant bacteria were recovered by scraping the Transwell wells with 1 mL tips and centrifugation (5 min at 5 000 rpm). Resistant bacteria were serially diluted and plated on TY agar plates, incubated for 48 h at 37°C under anaerobic conditions.

### *In vitro* biofilm assays

*C. difficile* ON cultures were diluted to a final *λ*_600 nm_ of 0.02 into fresh equilibrated Gut Microbiota Medium (GMM)^38^ or GMM supplemented with mucin, 1 mL per well was deposited in 24-well polystyrene tissue culture-treated plates (Falcon Clear Flat Bottom) and the plates were incubated at 37°C in anaerobic environment for 48 h. Type II mucin (Sigma M2378) and native mucin extracted from pork were diluted in Milli-Q water (concentration 40 mg/mL), autoclaved (15 min, 121°C) and added to the pre-equilibrated medium (final concentration 2 mg/mL). Biofilm biomass was measured using established methods.^39^ Briefly, spent media was removed. Biofilms were air dried and stained with crystal violet (CV; 0.2% w/v) for 10 min. CV was removed by inversion; wells were washed twice with PBS and then air-dried. Dye bound to the biofilm biomass was solubilized by adding 1 mL of 75% (%v/v) ethanol and the absorbance, corresponding to the biofilm biomass, was measured at a *λ*_600 nm_ with a plate reader (Promega GloMax Explorer). When needed, the solubilized dye was diluted with 75% ethanol for the reading to remain in the linear range. Sterile GMM or GMM with mucin was used as a blank for the assays.

### CDT toxin assays

The two CDT subunits, CDTa and activated CDTb, were generated, as previously described, using an *E. coli* expression system.^40^ Briefly, the complete ORFs of CDTa and CDTb were amplified by PCR from genomic DNA of *C. difficile* strain R20291 (GenBank: FN545816.1). Only the sequences bp 127–389 for CDTa and bp 127–2628 for CDTb (without the leader sequences) were cloned into the pGEX-2T vector to genetically engineer the GST fusion proteins of the mature CDTa and CDTb. GST – CDTa and GST – CDTb were expressed in *E. coli* following a standard protocol. Gene expression was induced by 100 μM isopropyl-β-D-thiogalactopyranosid when the bacterial cultures reached an OD_600 nm_ of 0.6. The GST fusion proteins were affinity purified via glutathione-sepharose (GE Healthcare, Dornstadt, Germany) by gravity flow, and the proteins were released either by thrombin (0.06 U/μg protein, 4°C ON for CDTa) or by elution with 10 mm glutathione (CDTb). Eluted GST – CDTb was directly activated by trypsin (0.2 μg/μg protein, 30 min at RT). Trypsin was inactivated by 2 mm 4-(2-Aminoethyl) benzenesulfonyl fluoride, and the solution was dialyzed against PBS ON.

### ELISA-based measurement of CDT

A 96-well immuno-plate (Nunc Maxisorp) was coated ON with CdtB capture antibody (MBS396782, MyBioSource) diluted into PBS. Plates were washed twice (PBS +1% Tween 20). Blocking buffer (PBS +2% BSA) was added, and plates were incubated for at least 1 h at RT and washed twice. Bacterial supernatants or lysates were serially diluted in PBS and incubated in coated plates for 90 min at RT. After two washes, chicken anti-CdtB IgY HRP conjugated antibody (MBS396785, MyBioSource) was added for 1–2 h at RT. The wells were washed four times and incubated with TMB (3,3′,5,5′tetramethylbenzidine) HRP substrate solution (Thermo Fisher Scientific) for 5–30 min in the dark. The stop solution (H_2_SO_4_; 0.2 M) was added into each well and the absorbance of the reaction was read at 450 nm (Promega Glomax Explorer plate reader).

### ELISA-based measurement of TcdA

Total TcdA amount was quantified from supernatants. Briefly, 1.5 mL of culture was harvested by centrifugation for 4 min at 13 000 rpm. Supernatants were collected, and the bacterial pellets were frozen at −20°C. The supernatant fractions were then analyzed by ELISA. A 96-well immuno-plate (Nunc Maxisorp) was coated with 2 μg/mL of anti-toxin A rabbit polyclonal antibody (Abcam, Inc.) ON at 4°C. The coated wells were washed and incubated with Superblock blocking buffer (Thermo Fisher Scientific) for 1 h. The wells were then washed and air-dried. Samples were added into the wells, and the plate was incubated at 37°C for 90 min. After washings, 0.2 μg/mL of an anti-toxin A chicken horseradish peroxidase (HRP) antibody (LSBio) was added in each well, and the plate was incubated for 1 h at 37°C. The wells were washed and incubated with a TMB (3,3′,5,5′tetramethylbenzidine) substrate solution (Thermo Fisher Scientific) for 15 min in the dark. The stop solution (H_2_SO_4_; 0.2 M) was added into each well, and the absorbance of the reaction was read at 450 nm (Promega Glomax Explorer plate reader).

### RNA isolation and quantitative reverse-transcriptase PCR

Cells were washed once with PBS (Gibco), lysed in RLT buffer (Qiagen) and freeze at −80°C until extraction was performed. RNA was extracted with RNeasy mini Kit (Qiagen) following manufacturer’s recommendations. DNA digestion was carried out in columns using RNase-free DNase set (Qiagen) and RNA clean-up with a RNeasy MinElute Cleanup kit (Qiagen). The RNA yield was measured with Nanodrop. cDNA was obtained with QuantiTect Reverse Transcription Kit (Qiagen) following manufacturer’s instructions. The quantitative Real-Time PCR was performed on StepOne Real-Time PCR Systems (Thermo Scientific) using SsoFast EvaGreen Supermix (Bio-Rad) following manufacturer’s instructions. Each reaction was performed in technical triplicate with two or three independent biological replicates. Data were analyzed by the ΔΔCt method. Gene expression levels were normalized to the *rps13* gene.

### Spore preparation

Spore suspensions were prepared as previously described.^41^ Briefly, 200 μl from ON cultures of *C. difficile* strains were plated on sporulation medium for *C. difficile* (SMC) medium (9% Bacto peptone, 0.5% proteose peptone, 0.15% tris base, and 0.1% ammonium sulfate) and were incubated at 37°C for 7 days under anaerobic conditions. Spores were scraped off and resuspended in 2 mL of sterile ice cold water and incubated for 7 days at 4°C. Cell fragments and spores were separated by centrifugation using a HistoDenz (Sigma-Aldrich) gradient.^42^ Spores were enumerated on TY supplemented with 1% taurocholate and kept at 4°C on glass vials.

### Ethics statement

Animal studies were performed in agreement with European and French guidelines (Directive 86/609/CEE and Decree 87–848 of October 19, 1987). The study received the approval of the Institut Pasteur Safety Committee (Protocol n°18086) and the ethical approval of the local ethical committee “Comité d’Ethique en Experimentation Animale Institut Pasteur no. 89 (CETEA)” (CETEA dap190131).

### Germ-free mice infection experiments

C57/BL6 7-week-old gnotobiotic male and female mice from Institut Pasteur Animal facilities were challenged with *C. difficile* spores (2 × 10^3^ per mice) by oral gavage. To assess bacterial persistence, fecal pellets were collected over a 13-day period (days 0, 1, 2, 6, 7, 8, 9, and 13). Fecal pellets were homogenized in the anaerobic hood in 1 mL of PBS, serially diluted, and plated in triplicate on BHI agar containing 2% defibrinated horse blood, 0.1% taurocholate, tetracycline (5 μg/mL), ciprofloxacin (5 μg/mL) cefoxitin (8 μg/mL), and cycloserine (250 μg/mL) to assess the total number of CFUs. To assess the total number of spores, diluted fecal pellets were incubated in ethanol (50% v/v final concentration) for at least 1 h and plated in triplicates using the same medium.

### Measurement of lipocalin-2 intestinal levels

Frozen fecal samples were reconstituted in PBS and vortexed for 5 min to homogenize the fecal suspension. Then samples were centrifuged for 10 min at 10 000 rpm and 4°C. Clear supernatants were collected and stored at −20°C until analysis. Lcn-2 levels were estimated in the supernatants using Duoset murine Lcn-2 ELISA kit (R&D Systems). Samples from day 0 (before infection) were used as negative controls.

### Histological processing and staining of tissue samples

Intestinal tissues were recovered, and full rolls were placed in Carnoy’s fixative solution (60% ethanol, 30% chloroform, and 10% glacial acetic acid) ON at 4°C. Later, ethanol gradients were applied to wash fixed tissues (70%, 80%, 95%, and 100% vol/vol). Tissues were embedded in ethanol/xylene (1:1) and xylene, followed by embedding in Paraffin. Tissue blocks were laterally sectioned at 10 μm and were stained with hematoxylin and eosin (HE) or Periodic Acid Schiff (PAS) as previously described^43^ to assess histological score or perform immunostainings.

## Quantification and statistical analysis

### Image analysis of *C.* *difficile* biofilms in the TIM and IoC models

TIM and IoC images were analyzed using the same analysis scripts, developed in Python.^44^ First, 3D images were projected along Z to yield 2D multi-channel images. For each image, chromatic aberration was corrected by registering the bacteria channel with respect to the mucin channel, using phase cross-correlation^45^ implemented in scikit-image.^46^ The mucin signal was quantified by first segmenting the tissue surface in the image, on the nuclei channel combined with the actin channel, using an intensity threshold. The mean mucin signal and its standard deviation were then measured within the resulting tissue mask. Bacteria were segmented in the far-red channel using Omnipose.^47^ A bacterium was classified as positive for mucin if the mean mucin intensity within the bacteria mask was larger than the mean mucin signal in the tissue plus the standard deviation. The count of all mucin-positive and negative bacteria and their total surface were then reported for each image. Results were exported to ImageJ TIFFs and ImageJ ROIs with the tifffile tool^48^ and were manually inspected using Fiji.^49^

### Measurement of mucin thickness and goblet cells in the colon of infected mice

Colonic sections were also stained with Alcian Blue, preferentially staining mucopolysaccharides, and 40 crypts were randomly selected per animal to determine goblet cell number per crypt.

### Image analysis of *C.* *difficile* biofilms in the colon and cecum of infected mice

Colon and cecum images were analyzed like TIM and IoC models with minor modifications (see above). Bacteria were segmented as a mask and not as single bacteria by thresholding after filtering by a 9 × 9 median filter and a gaussian filter with σ = 0.5 pixels.

### Statistical analysis

Statistical significance was determined using unpaired *t* tests or multiple unpaired Holm-Sidak t tests. For multiple comparisons, analysis of variance (ANOVA) was used with Bonferroni’s, Dunnett’s or Geisser-Greenhouse correction as recommended. Mann Whitney tests were performed for biofilms, mucin thickness, and goblet cell analyses. Statistics were completed using Prism 8.0 (GraphPad Software). Specific details with regard to statistical tests, statistical significance values (“p”), sample sizes (‘‘n’’) and replicates are indicated in the figure legends. For all analysis, significance was considered as *p* < 0.05.

### Data and code availability

- The full code for the image analysis performed in this paper is available publicly at https://gitlab.pasteur.fr/iah-public/clostridioides-difficile-binary-toxin-cdt-induces-biofilm-like-persisting-microcolonies
- Image data is available upon reasonable request.

## Results

### CDT induces mucin-associated microcolonies

Previous research has focused on CDT-induced clostridia adherence to epithelial cells by exposing cell monolayers to purified binary toxin and *C. difficile* cells for short periods of time.^15^ However, the long-term effects of this toxin have not been explored. To address this gap, we standardized two hypoxic cell culture models: a 2D Transwell Intestinal Model (TIM) with polarized cells under static conditions and a 3D Intestine-on Chip (IoC) microfluidic system that mimics flow and peristaltic intestinal motions. Both models were established with Caco-2 cells alone or Caco-2 cells co-cultured with HT29-MTX cells (a mucus secreting cell-line) under hypoxia conditions (4% O_2_ and 5% CO_2_) optimized to maintain the viability of both eukaryotic and *C. difficile* cells (Supplemental Fig. S1A and S1C). Cell morphology, structure, and mucus production were assessed using appropriate markers (Supplemental Fig. S1B and S1D). Eukaryotic cell cytotoxicity was monitored with lactate dehydrogenase (LDH) release assays (Supplemental Fig. S1E and S1F).

To evaluate the role of CDT in *C. difficile* colonization, two different strains were used: the reference strain 630Δ*erm* (TcdAB^+^CDT^−^) and the epidemic NAP1/B1/027 strain UK1 (TcdAB^+^CDT^+^). To assess the role of CDT independently of TcdA and TcdB cytotoxic effects, we generated *in-frame tcdBEA* deletion mutants in 630Δ*erm* (630 ToxAB^−^CDT^−^) and UK1 (UK1 ToxAB^−^CDT^+^) strains (Supplemental Fig. S2A), as well as an *in-frame cdtAB* deletion mutant in the UK1 ToxAB^−^ background (UK1 ToxAB^−^CDT^−^) (Supplemental Fig. S2A). Deletion of either PaLoc or CdtLoc genes or both had no impact on *C. difficile* growth (Supplemental Fig. S2B). Additionally, we confirmed the absence of TcdA or CDT in culture supernatants from ToxAB^−^ and CDT^−^ strains (Supplemental Fig. S2C and S2D).

**Figure 1.**
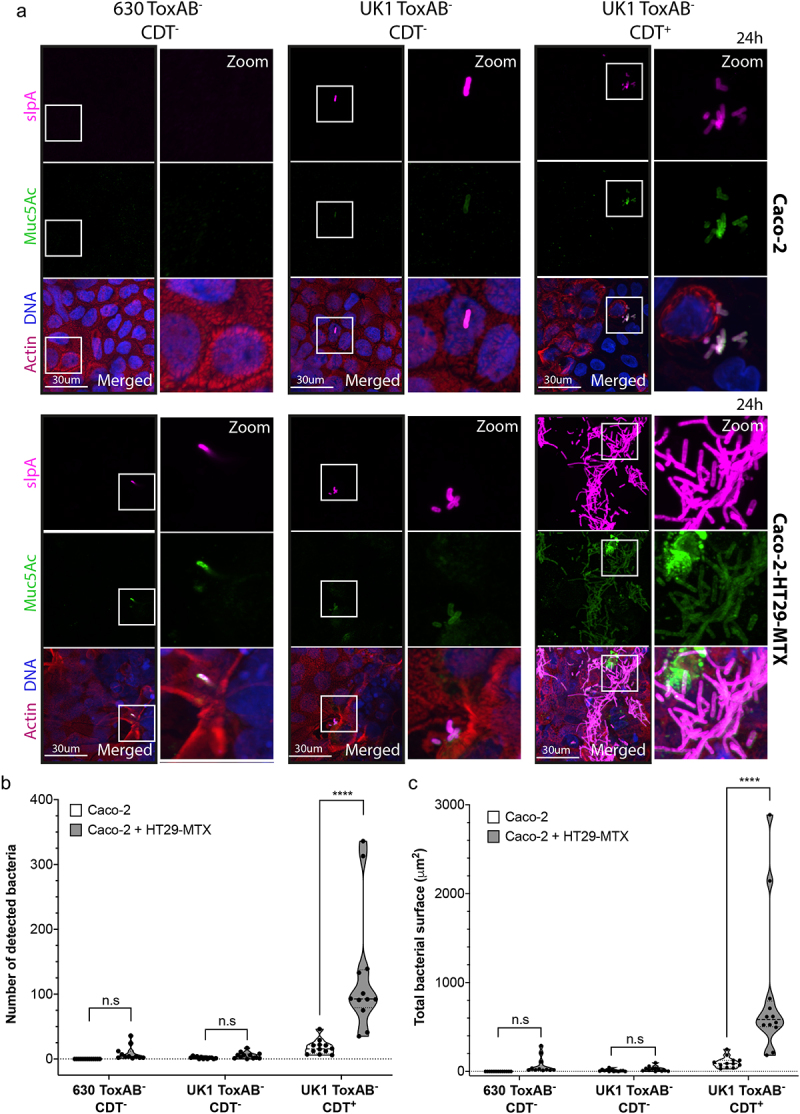
*C. difficile* forms CDT-mediated microcolonies in a Transwell Intestinal Model (TIM model) at 24 h p.i. (a) Representative 3D reconstructed images of Caco-2 cells alone or with HT29-MTX cells infected with 630 ToxAB^−^CDT^−^, UK1 ToxAB^−^CDT^−^, or UK1 ToxAB^−^CDT^+^ during 24 h under hypoxic conditions (4% O_2_, 5% CO_2_). DNA was labeled with DAPI (blue), mucin with anti-muc-5AC AF488 (green), actin with phalloidin rhodamine (red) and *C. difficile* with anti-SlpA^35^ AF647 (magenta). (b) Number of bacteria detected 24 h p.i in Caco-2 cells alone or with HT29-MTX cells infected with different *C. difficile* strains as indicated. (c) Total bacteria surface detected 24 h p.i in Caco-2 cells alone or with HT29-MTX cells infected with different *C. difficile* strains as indicated. The number of bacteria and total bacterial surface detected are reported for each image and at least 10 images were quantified per condition. Each black circle in the graph represents one image. Data and quantifications are representative of three independent biological replicates. Multiple unpaired *t* tests were performed and statistical significance is represented with ****(*p* < 0.0001).

Caco-2 cells alone or co-cultured with HT29-MTX cells in the TIM model were first infected with *C. difficile* mutants 630 ToxAB^−^CDT^−^, UK1 ToxAB^−^CDT^+^ and UK1 ToxAB^−^CDT^−^. Immunofluorescence (IF) microscopy was carried out to visualize *C. difficile* cells. An anti-SlpA monoclonal antibody was used to label *C. difficile* ^35^ and mucin was labeled with an anti-Muc-5AC antibody (Figure 1a). Whereas only few bacteria CDT^−^ were detected on Caco-2 cells co-cultured with the HT29-MTX, a large number of CDT^+^ bacteria were observed, organized as microcolonies or clumps and colocalizing with Muc-5AC (Figure 1a). Compared to Caco-2 cells alone, a greater number of bacteria were observed on Caco-2 cells co-cultured with HT29-MTX cells at 24 h post infection (p.i.) indicating that the presence of mucin producing-cells stimulates *C. difficile* adhesion (Figure 1a and Supplemental Fig. S3A). A quantitative approach revealed that the number of bacteria (up to 300) and the total surface of bacteria (200–3000 µm^2^) increased up to 10 times in the presence of CDT and mucin-producing cells (Figure 1b,c), suggesting a 3D biofilm-like structure.

In addition, viable vegetative cells (CFU) and spores were numerated 24 h p.i. (Supplemental Fig. S4A and S4B). The number of viable cells was similar between UK1 CDT^−^ and CDT^+^, with a 2-log increase at 24 h, whereas no CFU increase was observed for 630 CDT^−^ (inoculum 10^6^ bacteria/mL) (Supplemental Fig. S4A). Since no difference in CFU was observed between Caco-2 cells alone or co-cultured with HT29-MTX, *C. difficile* adhesion was monitored only in Caco-2 cells co-cultured with HT29-MTX at different time points in the TIM model. The 630 strain adhered much less than the two UK1 mutant strains and the UK1 CDT^+^ strain showed significantly better adhesion than the CDT^−^ isogenic strain at 24 h p.i. (Supplemental Fig. S4C).

**Figure 2.**
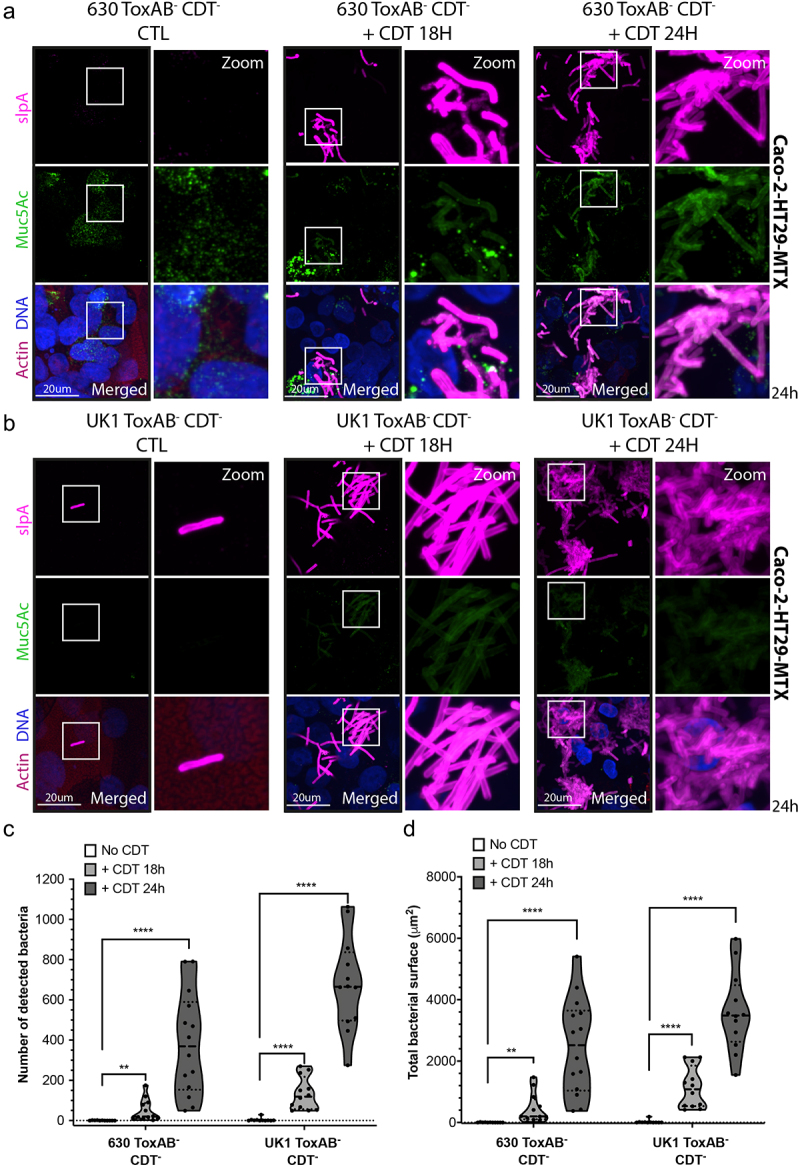
Purified CDT toxin induces microcolonies formation in CDT^−^ strains in the Transwell Intestinal Model. Representative 3D reconstructed images of Caco-2 cells cocultured with HT29-MTX cells infected with (a) 630 ToxAB^−^CDT^−^ or (b) UK1 ToxAB^−^CDT^−^ during 24 h under hypoxic conditions (4%O_2_, 5%CO_2_). Infected intestinal cells were exposed to CdtA (200 ng/mL) and activated CdtB (400 ng/mL) during 18 h or 24 h. DNA was labeled with DAPI (blue), mucin with anti-Muc5AC AF488 (green), actin with phalloidin rhodamine (red) and *C. difficile* with anti-SlpA^35^ AF647 (magenta). (c) Number of bacteria detected 24 h p.i in Caco-2 cells cocultured with HT29-MTX cells infected with *C. difficile* strains as indicated. (d) Total bacteria surface detected 24 h p.i in cells infected with *C. difficile* strains as indicated. The number of bacteria and total bacterial surface detected are reported for each image and at least 10 images were quantified per condition. Each black circle in the graph represents one image. Data and quantifications are representative of three independent biological replicates. Multiple unpaired *t* tests were performed and statistical significance is represented with **(*p* ≤ 0.01), ****(*p* ≤ 0.0001).

To validate that mucin-associated microcolonies formation is specifically induced by the CDT toxin, we performed a rescue experiment in the TIM model. We infected Caco-2 cells co-cultured with HT29-MTX cells with UK1 or 630 ToxAB^−^CDT^−^ strains and exogenously supplied the purified CDT toxin^40^ at 6 h p.i or at the time of the infection, corresponding to 18 and 24 h treatment, respectively. The addition of purified CDT resulted in the formation of strong microcolonies for both CDT^−^ strains (Figure 2a,b). The number of detected bacteria (up to 300 bacteria) and the microcolony surface (up to 2000 µm^2^) observed after 18 h of CDT treatment (Figure 2c,d) were similar to those obtained with UK1 CDT^+^ at 24 h p.i. (Figure 1b,c). When CDT was added at 18 h p.i (6 h treatment) bacteria were not able to form microcolonies (Supplemental Fig. S5A and S5B). Cell exposure to CDT during 24 h dramatically increased the number of adhered bacteria (up to 1000 bacteria) and the surface of the microcolonies (up to 6000 µm^2^) (Figure 2c,d). The formation of similar mucin-associated microcolonies by the strain 630, naturally lacking the CDT toxin, provides robust evidence that CDT is the sole factor involved in their formation. Altogether, these results strongly support that CDT toxin increases bacterial adhesion in the presence of mucin and allows subsequent formation of microcolonies. These data suggest that the CDT-mediated microcolonies could contribute to *C. difficile* gut colonization.

### CDT-dependent 3D microcolonies favor *C* *difficile* colonization

**Figure 3.**
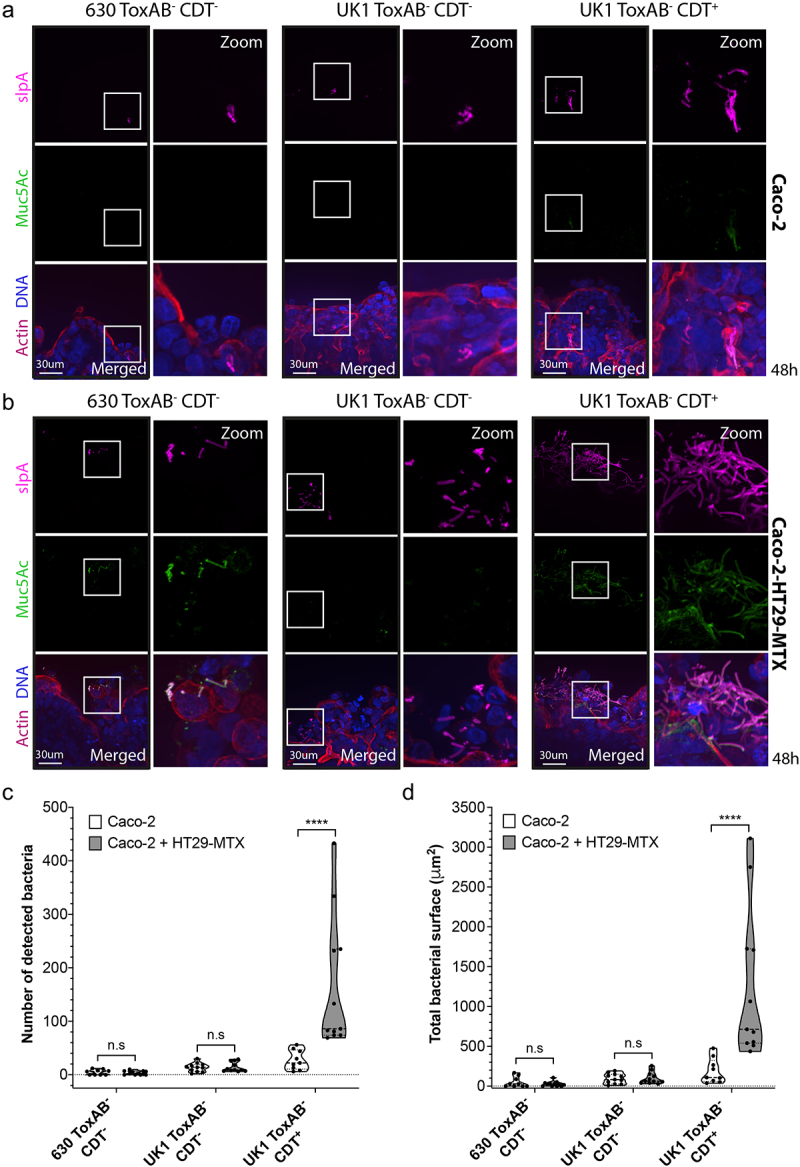
*C. difficile* forms CDT-mediated microcolonies in an intestine-on-chip (IoC) model at 48 h p.i. Representative 3D reconstructed images of (a) Caco-2 cells alone or (b) Caco-2 cells cocultured with HT29-MTX cells infected with 630 ToxAB^−^CDT^−^, UK1 ToxAB^−^CDT^−^ or UK1 ToxAB^−^CDT^+^ during 48 h under hypoxic conditions (4% O_2_, 5% CO_2_). DNA was labeled with DAPI (blue), mucin with anti-Muc5AC AF488 (green), actin with phalloidin rhodamine (red) and *C. difficile* with anti-SlpA AF647 (magenta). (c) Number of bacteria detected 48 h p.i in Caco-2 cells alone or with HT29-MTX cells infected with *C. difficile* strains as indicated. (d) Total bacteria surface detected 48 h p.i in Caco-2 cells alone or with HT29-MTX cells infected with *C. difficile* strains as indicated. The number of bacteria and total bacterial surface detected are reported for each image and at least 10 images were quantified per condition. Each black circle in the graph represents one image. Data represents mean with SEM and quantifications are representative of two independent biological replicates. Multiple unpaired *t* tests were performed and statistical significance is represented with ****(*p* < 0.0001).

To better decipher the role played by CDT in microcolony formation and *C. difficile* gut colonization, we next used the IoC model cultured with Caco-2 cells alone or cocultured with HT29-MTX cells. Upon the development of the 3D intestinal structure (6–7 days in normoxic conditions), the IoC chips were placed in hypoxia (4% of O_2_) and infected with 630 ToxAB^−^CDT^−^, UK1 ToxAB^−^CDT^−^ or UK1 ToxAB^−^CDT^+^. IF analyses revealed the formation of 3D microcolonies in the IoC model only with Caco-2 cells co-cultured with HT29-MTX cells at 24 and 48 h p.i with the CDT^+^ strain but not with the CDT^−^ strains (Figure 3a,b, Supplemental Fig. S6A and S6B). CDT^+^ strains showed a higher count of bacteria and a more extensive bacterial surface compared to the CDT^−^ strains (Figure 3c,d, Supplemental Fig. S6C and S6D). The number of bacteria and the surface of microcolonies observed with the IoC model at 48 h p.i., although slightly lower, were consistent with those obtained with the TIM model after 24 h of infection (Figure 1b,c). Moreover, the microcolonies observed in IoC colocalized with mucin as in the TIM model (Supplemental Fig. S3A and S3B). The delay in microcolony formation in the IoC model when compared with the TIM can be explained by the presence of flow. Nonetheless, the IoC model confirms that CDT promotes *C. difficile* colonization through the formation of 3D mucin-associated microcolonies.

### CDT mucin-associated microcolonies possess biofilm-like properties

**Figure 4.**
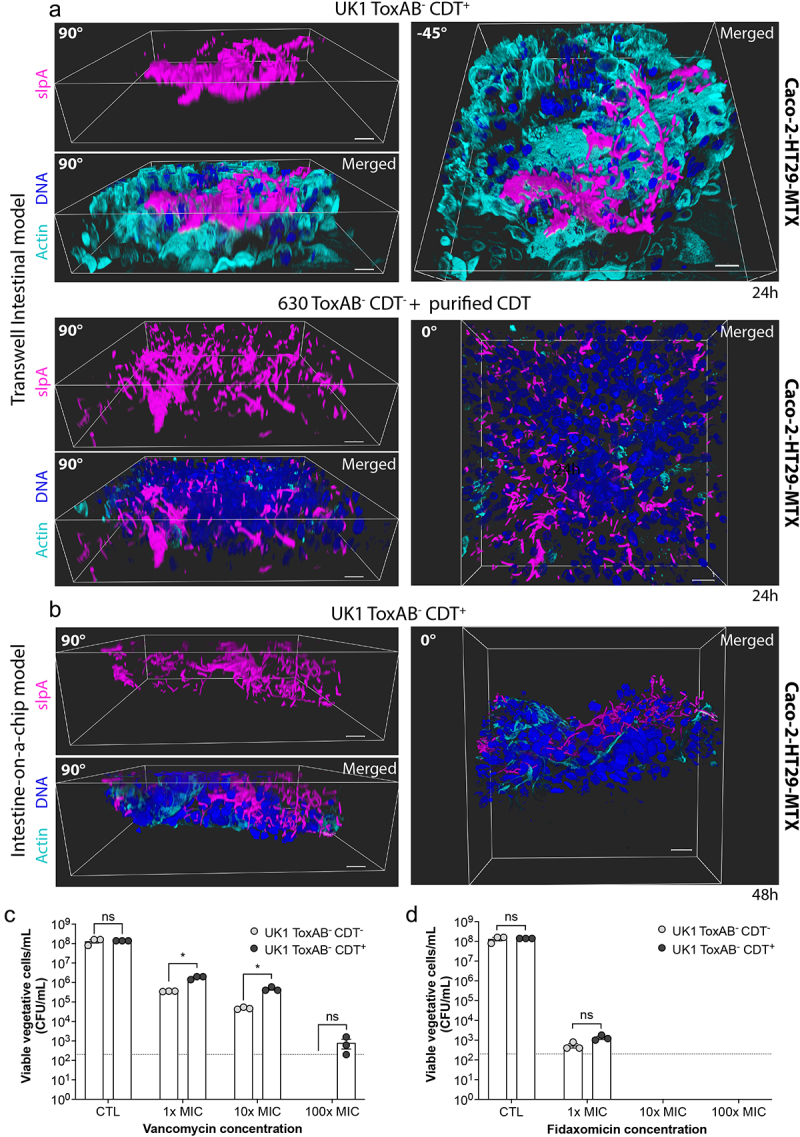
CDT mucin-associated microcolonies possess biofilm-like properties Representative 3D images reconstructed with blend mode. Caco-2 cells cocultured with HT29-MTX cells in the TIM (a) or IoC model (b) were infected with UK1 ToxAB^−^CDT^+^ or 630 ToxAB^−^CDT^−^ and exposed to CdtA (200 ng/mL) and activated CdtB (400 ng/mL) during 24 h or 48 h as indicated under hypoxic conditions (4%O_2_, 5%CO_2_). DNA was labeled with DAPI (blue), actin with phalloidin rhodamine (cyan) and *C. difficile* with anti-SlpA AF647 (magenta). Scale bars represent 20 µm. (c,d) Caco-2 cells cocultured with HT29-MTX cells in the TIM model were infected with UK1 ToxAB^−^CDT^−^ and UK1 ToxAB^−^CDT^+^, 24 h p.i infected cells were treated with different concentrations of vancomycin (c) or fidaxomicin (d) for additional 24 h. Viable vegetative cells were recovered 48 h p.i. The vancomycin and fidaxomicin concentrations used were 1×, 10×, and 100× times higher than the MIC. Data represents mean with SEM from three independent biological replicates. Multiple unpaired *t* tests were performed and statistical significance is represented with * (*p* < 0.05).

Previous observations have indicated that *C. difficile* biofilm-like structures are located adjacent to epithelial cells and cause damages and cell death in the microvilli structures.^50^ Additionally, recent studies have reported that *C. difficile* forms complex biofilms with porous and fiber-like structures, comprising dead cells, abundant eDNA, proteins, and polysaccharides.^39,51,52^ To further characterize CDT-induced biofilm-like structures, IF microscopy was performed from the TIM and IoC models infected with the CDT^+^ strain 24 h or 48 h p.i., respectively, or the TIM model infected with the CDT^−^ strain incubated with the CDT toxin, 24 h p.i., and 3D images were reconstructed (Figure 4a,b). 3D structures of CDT-dependent biofilm-like were observed adjacent to epithelial cells, exhibiting porous and complex organization (Figure 4a,b). However, fiber-like polysaccharide structures were not revealed when IF was performed with an anti-PSII antibody known to detect *C. difficile* biofilm matrix in the TIM and IoC models (Fig S7A and 7B). Given that fiber-like structures are typically observed after 3 days of incubation,^39^ the possibility that these structures form in more mature biofilms cannot be excluded.

Bacteria embedded in biofilms exhibit increased resistance to antibiotics, antimicrobial peptides, and oxidative stresses.^53,54^ *In vitro*, *C. difficile* biofilms display better survival than planktonic cells when exposed to antibiotics commonly used to treat CDI, including vancomycin.^39,51^ *C. difficile* biofilms can withstand vancomycin concentrations up to 25 times higher than the Minimal Inhibitory Concentration (MIC).^55^ On the other hand, fidaxomicin, an effective antibiotic against CDI and known to decrease rCDI, is effective in disrupting *C. difficile* biofilms.^55,56^ Since CDT is a virulence factor associated with higher relapse rates,^22^ we hypothesized that CDT-induced microcolonies could present biofilm-like properties, enabling better resistance of *C. difficile* to vancomycin but not fidaxomicin. No difference in the MIC of fidaxomicin and vancomycin against the planktonic cells of UK1 ToxAB^+^CDT^+^, UK1 ToxAB^−^CDT^−^ and UK1 ToxAB^−^CDT^+^ strains grown in ADMEM medium was observed (Table S3), indicating that toxin gene deletions have no impact on resistance to these antibiotics. Caco-2 cells co-cultivated with HT29-MTX cells in the TIM model were then infected with UK1 ToxAB^−^CDT^−^ and UK1 ToxAB^−^CDT^+^ strains. After 24 h infection, cells were treated with different concentrations of vancomycin or fidaxomicin (1×, 10×, and 100× MIC) and incubated for an additional 24 h before counting viable CFU. No difference in CFU was observed between the two strains in the control condition with no antibiotic treatment (CTL in Figure 4c,d). However, the CDT^+^ strain was significantly more resistant to vancomycin than the CDT^−^ strain for all concentrations tested (Figure 4c). Whereas no viable CDT^−^ bacteria were detected with the highest vancomycin concentration, CDT^+^ bacteria were still present at a concentration of 10^3^ CFU/mL (Figure 4c). In contrast, fidaxomicin similarly impacted the viability of the CDT^+^ and CDT^−^ strains with a strong reduction of CFU at 1× MIC and no bacteria were detected at 10 or 100× MIC concentrations (Figure 4d). This result is in agreement with the effectiveness of fidaxomicin in eradicating *C. difficile* biofilms.^55,56^ Altogether, our data indicates that CDT mucin-associated microcolonies possess biofilm-like properties by resisting to vancomycin but not fidaxomicin.

### Mucin induces *C. difficile* biofilm formation *in vitro* and increases CDT levels

**Figure 5.**
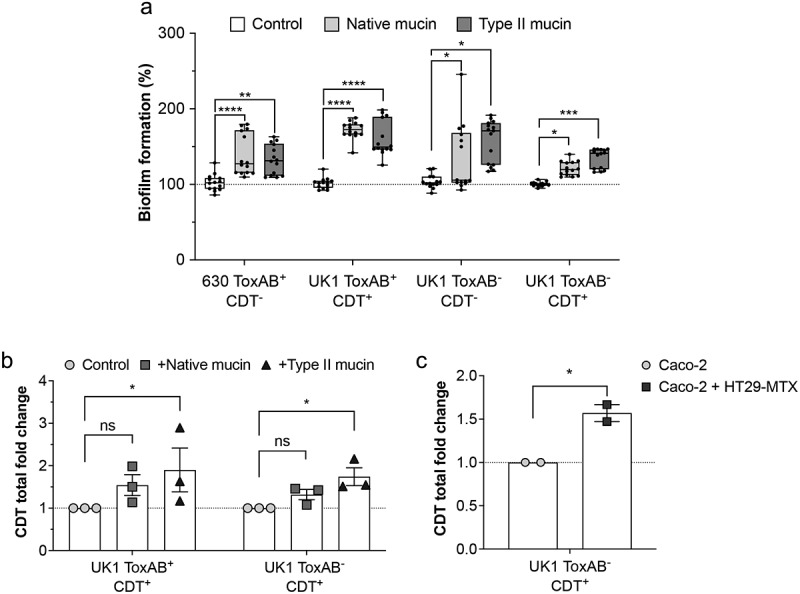
Mucin triggers *C. difficile* biofilm formation *in vitro* increasing CDT levels. (a) Biofilm formation evaluated by crystal violet biofilm assay. Strains were grown during 48 h in GMM medium alone (control) or with native mucin from pork or mucin type II. The mean OD of biofilm from strains grown in GMM alone is adjusted to 100%. Minimum–maximum boxplot shows three independent biological replicates with 14–15 technical replicates (black circles). Mann–Whitney U test was performed and statistical significance is represented (*p < 0.05, **p < 0.01, ***p < 0.001, ****p < 0.0001). (b) CDT toxin ELISA represented as total CDT fold change. Strains were grown during 48 h in GMM medium alone (control) or GMM with native or mucin type II. The level of CDT assayed from crude extracts and supernatant were normalized to the OD_600 nm_ of bacteria cultures. Data represents mean with SEM from three independent experiments. A two-way ANOVA with Bonferroni correction was performed (ns: not statistically significant, *p < 0.05). (c) CDT toxin ELISA represented as total CDT fold change. CDT was measured from supernatants recovered 24 h p.i from Caco-2 cells alone or co-cultured with HT29-MTX cells in the TIM model infected with UK1 ToxAB^−^CDT^+^. Data represents mean with SEM from two independent biological experiments. An unpaired *t* test was performed and statistical significance is represented (*p < 0.05).

Induction of *C. difficile* biofilm by Muc2 in antibiotic-treated human fecal bioreactors has previously been reported.^57^ Since the biofilm formation in this study was evaluated with the CDT^+^ strain R20291, we next wondered whether Muc2-dependent biofilm formation *in vitro* was mediated by CDT. To assess the role of CDT in biofilm formation, we cultured the strains 630 ToxAB^+^CDT^−^, UK1 ToxAB^+^CDT^+^, UK1 ToxAB^−^CDT^+^ and UK1 ToxAB^−^CDT^−^ during 48 h in the Gut Microbiota Medium (GMM), a rich medium mimicking the intestinal environment,^38^ alone or with different types of mucins. Biofilm formation *in-vitro* was induced by the presence of native mucin and type II mucin in a CDT-independent manner (Figure 5a). These data differ from the data obtained with the TIM and IoC models where the presence of both CDT and mucin was required to form biofilm-like microcolonies. Our findings suggest that both mucin and CDT may induce *C. difficile* biofilm formation through different mechanisms.

We next explored the potential influence of mucin on CDT production. CDT levels were assessed by enzyme-linked immunosorbent assay (ELISA) from supernatants and pellets collected after 48 h of growth in GMM alone or with mucin. In both UK1 ToxAB^+^CDT^+^ and UK1ToxAB^−^CDT^+^ strains, CDT levels significantly increased in the presence of type II mucin (Figure 5b). Determination of the extracellular levels of CDT from supernatants of infected Caco-2 cells alone or co-cultured with HT29-MTX cells in the TIM model at 24 h p.i. revealed a similar increase induced by the presence of mucin producing-cells (Figure 5c). Altogether, our data indicate that CDT mucin-associated microcolonies possess biofilm-like properties, as they resist vancomycin but not fidaxomicin, and that the presence of mucin induces *C. difficile* biofilm formation and increases CDT extracellular levels.

### CDT decreases mucin-related gene transcription, number of goblet cells, and mucin thickness

**Figure 6.**
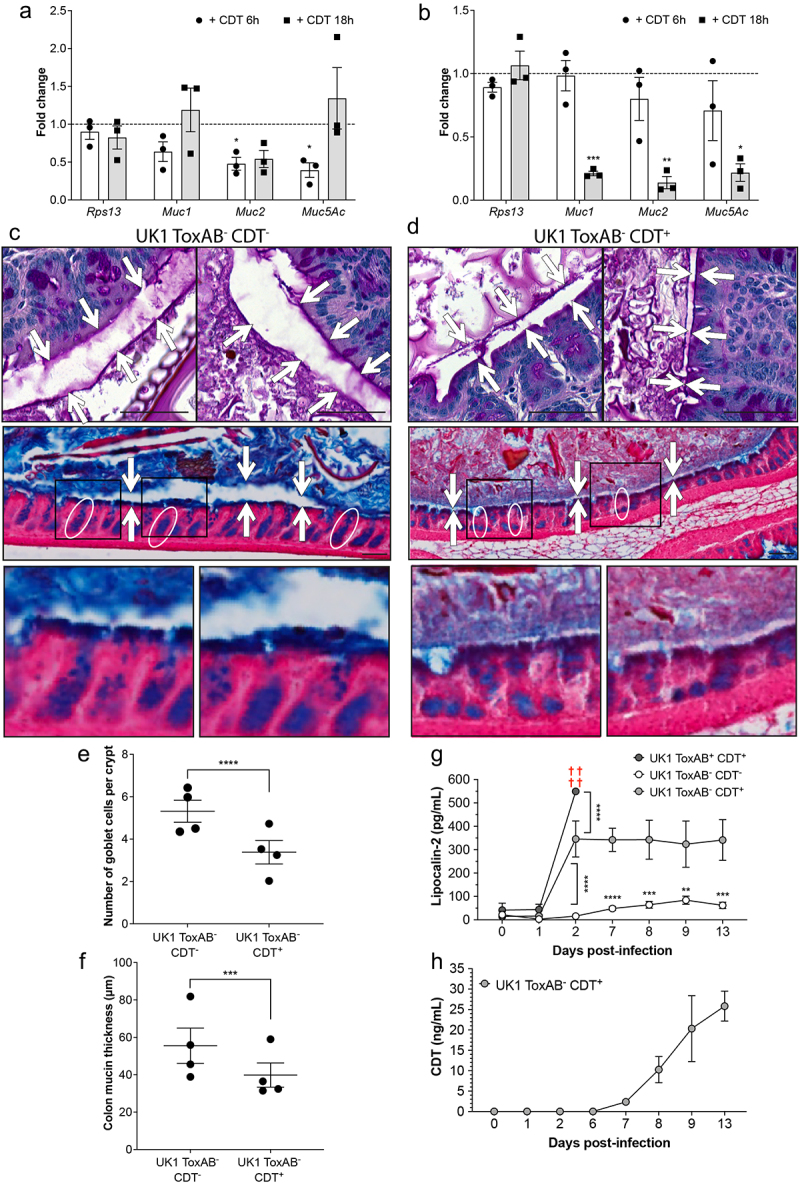
CDT decreases mucin-related gene transcription, number of goblet cells and mucin thickness. Caco-2 cells cocultured with HT29-MTX cells were treated or not with CdtA (200 ng/mL) and activated CdtB (400 ng/mL), and mRNA levels of treated or not treated cells were quantified by qRT-PCR in (a) the Transwell Intestinal Model (TIM) or (b) the Intestine-on-Chip model (IoC). Data represents mean with SEM from three independent biological replicates (TIM) or two independent biological replicates (IoC) with three technical replicates. Data is normalized to not treated cells and represented as fold change relative to the housekeeping gene *Rps13*. A two-way ANOVA with Geisser-Greenhouse correction test was performed. (c,d) Representative images of mucin thickness (white arrows) and goblet cells (circles) from mouse colonic sections infected with UK1 ToxAB^−^CDT^−^ (c) or UK1 ToxAB^−^CDT^+^ (d) and stained with HE (top) or PAS (bottom) 13 days p.i. Scale bars represent 50 µm. (e) Quantification of number of goblet cells per crypt from mouse colonic sections 13 days p.i. (*n* = 40 per mice, one point represents the mean value per mice) (f) quantification of mucin thickness from mouse colonic sections 13 days p.i. (*n* = 10 per mice, one point represents the mean value per mice). Mann Whitney tests were performed (E, F). (g) Fecal lipocalin-2 levels detected by ELISA before (day 0 and 1) and during *C. difficile* infection (day 2, 6, 7, 8, 9 and 13). Multiple unpaired *t* tests were performed. (h) Fecal CDT levels detected by ELISA before infection (day 0 and 1) and during *C. difficile* infection (day 2, 6, 7, 8, 9 and 13). Data represents mean with SEM and statistical significance is represented with *p < 0.05, **p < 0.01, ***p < 0.001 and, ****p < 0.0001. ns: no statistical significance.

*C. difficile* has previously been shown to adhere to human mucus and to decrease mucin secretion in enteroids.^58,59^ In addition, patients with CDI present decreased Muc2 levels and show alterations in mucin composition.^59^ We, therefore, aimed to determine whether the decreased mucin levels could be mediated by CDT. RNA were extracted from Caco-2 cells co-cultured with HT29-MTX cells treated with purified CDT (TIM and IoC models) and mucin mRNA levels were quantified by qRT-PCR (Figure 6a,b). Cells from CDT-treated TIM model showed a significant decrease of *Muc2* and *Muc5AC* mRNA abundance genes after 6 h but not 18 h of CDT treatment compared to untreated cells (Figure 6a). The impact of the CDT treatment was delayed in the IoC model, but a strong reduction of *Muc1*, *Muc2*, and *Muc5AC* mRNA abundance was observed after 18 h treatment (Figure 6b). The similar trend observed with both models indicates that CDT negatively regulates the mRNA abundance or stability of mucin-related genes.

To determine whether CDT influences *C. difficile* colonization and alters mucin levels *in-vivo*, C57Bl/6J germ-free mice were infected with spores from the UK1 ToxAB^+^CDT^+^, UK1 ToxAB^−^CDT^−^, or UK1 ToxAB^−^CDT^+^ strains. Mice infected with UK1 ToxAB^+^CDT^+^ were sacrificed 2 days p.i due to weight loss (Supplemental Fig. 8A and 8B), as typically observed.^60^ Assessment of the histological score revealed no significant difference in intestinal tissue between the CDT^+^ and CDT^−^ strains (Supplemental Fig. S7C). The number of total cells, vegetative cells, or spores detected in the feces of mice was similar for CDT^+^ and CDT^−^ strains during CDI (Supplemental Fig. S8D, S8E, and S8F). At day 8 p.i., mice infected with the CDT^−^ strain had cleared vegetative cells, while those infected with the CDT+ strain did not (Supplemental Fig. S7E). Overall, these results showed no difference in the number of vegetative cells released in the feces between CDT^+^ and CDT^−^ strains (Supplemental Fig. S8E). However, CDT^+^ infected mice showed a delayed clearance of *C. difficile* compared to CDT^−^ strain (Supplemental Fig. S8E). In addition, more spores and vegetative cells were found in the cecum of mice infected with the CDT^+^ strain at day 13 p.i. (Supplemental Fig. S8G), supporting a better persistence of the CDT^+^ strain in the cecum of mice.

Colon and cecum were retrieved and fixed with Carnoy to preserve mucin structure. Periodic Acid Schiff (PAS) staining of colon sections revealed a significant reduction in the number of goblet cells per crypt as well as mucin thickness in mice infected with the CDT^+^ strain compared to those infected with the CDT^−^ strain (Figure 6c–f). This result further supports the notion that CDT induces changes in mucin.

As toxin-induced inflammation is beneficial to *C. difficile* during infection,^61^ we sought to better understand the role of CDT in intestinal inflammation. To achieve this, we monitored the fecal lipocalin-2 (Lcn2) levels of mice infected with CDT^+^ and CDT^−^ strains. As expected, the strain ToxAB^+^CDT^+^ induced a strong inflammatory response 2 days p.i., whereas UK1 ToxAB^−^CDT^+^ and UK1 ToxAB^−^CDT^−^ strains showed intermediate or low levels of Lcn2, respectively (Figure 6g). Maximum inflammation occurred from day 2 p.i. with a significant contribution from CDT, which persisted until day 13 when the mice were sacrificed. Additionally, CDT levels in mice feces increased from 7 up to 13 days p.i., suggesting that CDT levels are persistent during CDI (Figure 6h). Collectively, these results demonstrate that CDT mediates changes in mucin thickness, decreases the number of goblet cells and induces an inflammatory response that is sustained throughout CDI.

### CDT toxin favors the formation of biofilm-like microcolonies in the cecum and colon of mice, increasing *C.* *difficile* persistence

**Figure 7.**
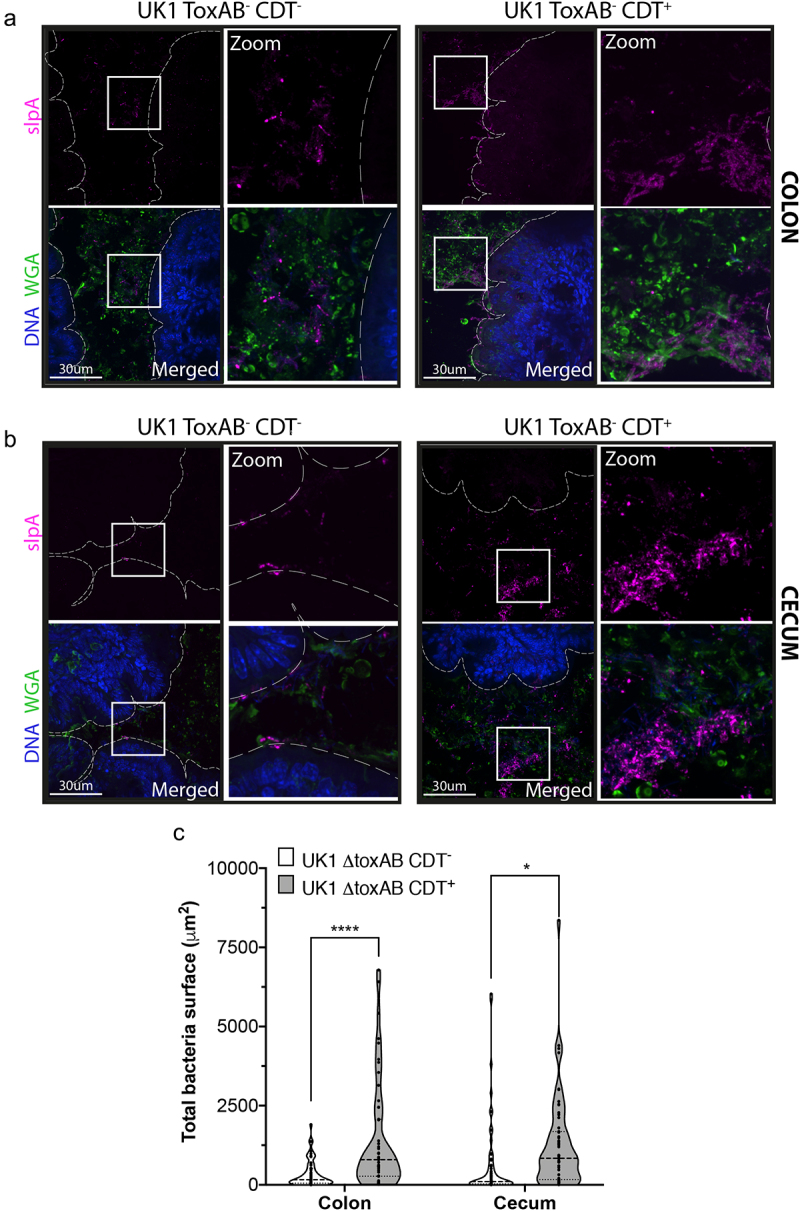
The CDT toxin promotes the formation of biofilm-like microcolonies in the cecum and colon of mice. (a) mouse colonic and (b) cecum sections were immunostained 13 days p.i. DNA was labeled with DAPI (blue), mucus layer with the lectin WGA AF488 (green), and *C. difficile* was labeled with anti-slpA AF647 (magenta). (c) Total bacterial surface per image was quantified from mice colonic and cecum sections 13 days p.i. Data and quantifications are representative of at least 13 images quantified per mice (four mice per condition, scale bar 30 µm). Each black circle in the graph represents one image (*n* ≥ 52). Multiple unpaired *t* tests were performed and statistical significance is represented (*p < 0.05, ****p < 0.0001).

In order to investigate whether CDT biofilm-like microcolonies were also formed *in vivo*, mice colonic sections and cecum sections were immuno-stained 13 days p.i. Interestingly, mucin-associated and embedded microcolonies were detected in the cecum and colon of mice infected with *C. difficile* (Figure 7a,b). Moreover, the total surface of these microcolonies in the colon and cecum was significantly higher in mice infected with CDT^+^ than in those infected with CDT^−^, suggesting that CDT promotes better colonization in colon and cecum (Figure 7c). The size of the CDT-microcolonies was similar in the cecum and colon with an average of around 1000 µm^2^ (Figure 7c). The presence of biofilm-like CDT-induced microcolonies in both the colon and cecum epithelium of mice underscores their biological importance and suggests that they may be involved not only in *C. difficile* colonization but also in *C. difficile* persistence.

## Discussion

We report here that purified or secreted *C. difficile* CDT binary toxin induces mucin-associated microcolonies *in vitro* .^62^ CDT-induced microcolonies enhance *C. difficile* resistance to vancomycin but not to fidaxomicin, consistent with biofilm structures. Biofilm-like microcolonies also form *in vivo* in the cecum and colon of mice, facilitating *C. difficile* colonization and potentially promoting *C. difficile* persistence in mice.^62^ In addition, we showed that the presence of mucin induces *C. difficile* biofilm formation and increases CDT levels. CDT, in turn, induces transcriptional changes of mucin-related genes resulting in a reduction of mucin thickness and goblet cells in the colon.

In our study, we used two models reflecting the intestinal architecture with mucus-producing cells infected with a *C. difficile* CDT^+^ or CDT^−^ strain incubated with purified CDT under hypoxic conditions. These experimental conditions revealed that CDT induced microcolonies with biofilm-like structure. We speculate that CDT activity, by inducing local modifications at the infection site, including on mucin and extracellular matrix, promote *C. difficile* attachment and cell growth, a prerequisite for *C. difficile* biofilm formation. Biofilms are associated with persistent infections^63^ due in part to the capacity of bacteria embedded inside biofilms to better resist to antibiotics.^53^ Formation of *C. difficile* mono-species biofilm has been shown *in vitro* ^64,65^ and is induced in response to sub-inhibitory concentrations of antibiotics or metabolites whose concentrations vary during gut dysbiosis.^51,52,65^ *C. difficile* is also found in multi-species biofilms formed by the gut microbiota, constituting a potential reservoir leading to asymptomatic carriage and risk of recurrent infection after antibiotic therapy.^51,64,66–68^ However, whether the formation of a *C. difficile* mono-species biofilm can trigger a multispecies biofilm remains an open question. Given that our study involved the use of a strain producing both CDTa^+^ and CDTb^+^, or purified extracts containing both subunits, an important remaining question is whether either CDTa or CDTb subunit alone can induce biofilm-like microcolonies with antibiotic-resistant properties.

Our data also showed that CDT expression is upregulated in the presence of mucin or mucin sugar derivates.^62^ However, the question of which specific mucin-derived monosaccharide(s) or polysaccharides(s) induce CDT expression remains open. In agreement with our data, CDT levels in patients are 20-fold higher than the CDT levels measured *in vitro* .^69^ The binary toxin CDT might be strategically used by *C. difficile* to establish a biofilm embedded inside the mucus layer. This biofilm may help *C. difficile* persist in the host, allowing it to receive nutrient, resist shear forces and flow^70^ and protect itself against antimicrobial agents,^71,72^ high oxygen tensions,^73^ bile salts, and oxygen radicals.^74,75^ Within the biofilm, the CDT-mediated alteration of mucus may also bring the toxins A and B closer to the epithelium cells, thus increasing CDI severity.^20,23^

The release of mucin and antimicrobial molecules is regulated by the commensal microbiota.^76^ Pathogens can also influence the secretion of mucin by goblet cells,^77–79^ glycosylation of mucins^80^ or reduce mucus viscosity.^81^ It has been shown that mucin facilitates *C. difficile* colonization^57–59,82,83^ and that *C. difficile* binds to mucus from mice and humans.^57,58,66,84,85^ Engevik *et al*. reported that *C. difficile* strain BAA-1878 decreases Muc2 secretion in human intestinal organoids (HIOs) but this change was not associated with the capacity of this strain to produce CDT.^59^ Our data provide evidence that CDT alters the level of intestinal mucus by a mechanism that remains to be investigated. Decreasing mucin transcription through CDT could be one of the mechanisms used by *C. difficile* to reach the gut epithelium. *C. difficile* uses in priority Stickland-acceptor amino acids such as serine and threonine *in vivo*,^60,86^ both being the main amino-acids of the mucin peptide backbone.^87^ Furtado *et al.*, recently showed that uptake of serine and threonine is upregulated in *C. difficile* in presence of mucus.^88^ Intriguingly, lack of threonine resulted in a decrease of the mucus layer and goblet producer cells.^89^ Therefore, we can speculate that *C. difficile* mucus-associated microcolonies result in a decrease of the threonine levels leading to a reduction of the mucus layer thickness and shrinkage of goblet cells.^90^

A limitation in our study is the utilization of the goblet cell line HT29-MTX due to the difficulties to obtain colon primary cells from human biopsies. HT29-MTX cells produce Muc5AC instead of Muc2. Muc2 and Muc5AC are, respectively, secreted gel-forming and polymer-forming mucins with similar domains.^91^ Muc2 is observed in goblet cells of the small and large intestinal mucosa, whereas Muc5AC is predominantly expressed in the gastric epithelium.^92^ Muc5AC is absent from the normal colon, but frequently present in colorectal adenomas and colon cancers.^93^ Further studies using HIOs will be necessary to elucidate the mechanism of mucin thickness reduction mediated by CDT.

Overall, we have identified a new role of CDT during later stages of CDI that contributes to a more comprehensive understanding of the association between CDT, the increased severity of CDI and higher rCDI.^4,18–24^ In addition to the increased adherence of CDT^+^ strains to host cells, which has previously been reported by Schwan *et al*.,^14,15^ this study demonstrates that CDT has a significant impact on the formation of biofilm-like microcolonies with antibiotic-resistance properties. Furthermore, our results indicate that these CDT induced biofilm-like microcolonies are implicated in *C. difficile* colonization and persistence, as evidenced by our organ-on-chip and mice experiments, respectively. This study opens new perspectives regarding the methods used by enteric pathogens to create a niche in the gut epithelium as a method to persist.

## Supplementary Material

Supplemental Material
