# Supplementary material for: Clostridioides difficile binary toxin CDT induces biofilm-like persisting microcolonies
Source: Gut Microbes. 2024 Dec 24;17(1):2444411. doi: 10.1080/19490976.2024.2444411 (PMC12931720; doi:10.1080/19490976.2024.2444411)
Supplement: Supplemental Material [file KGMI_A_2444411_SM5418.zip › Meza_Torres_et_al_Supplemental_Information_071124.docx]

***Clostridioides difficile* binary toxin CDT induces biofilm-like persisting microcolonies**

Jazmin Meza-Torres^1,2^*, Jean-Yves Tinevez^3^, Aline Crouzols^1^, Héloïse Mary^4,5^, Minhee Kim^4^, Lise Hunault^6^, Susan Chamorro-Rodriguez^1^, Emilie Lejal^7^, Pamela Altamirano-Silva^8^, Déborah Groussard^9^, Samy Gobaa^4^, Johann Peltier^7^, Benoit Chassaing^10,11^, & Bruno Dupuy^1^**

**SUPPLEMENTAL FIGURES**


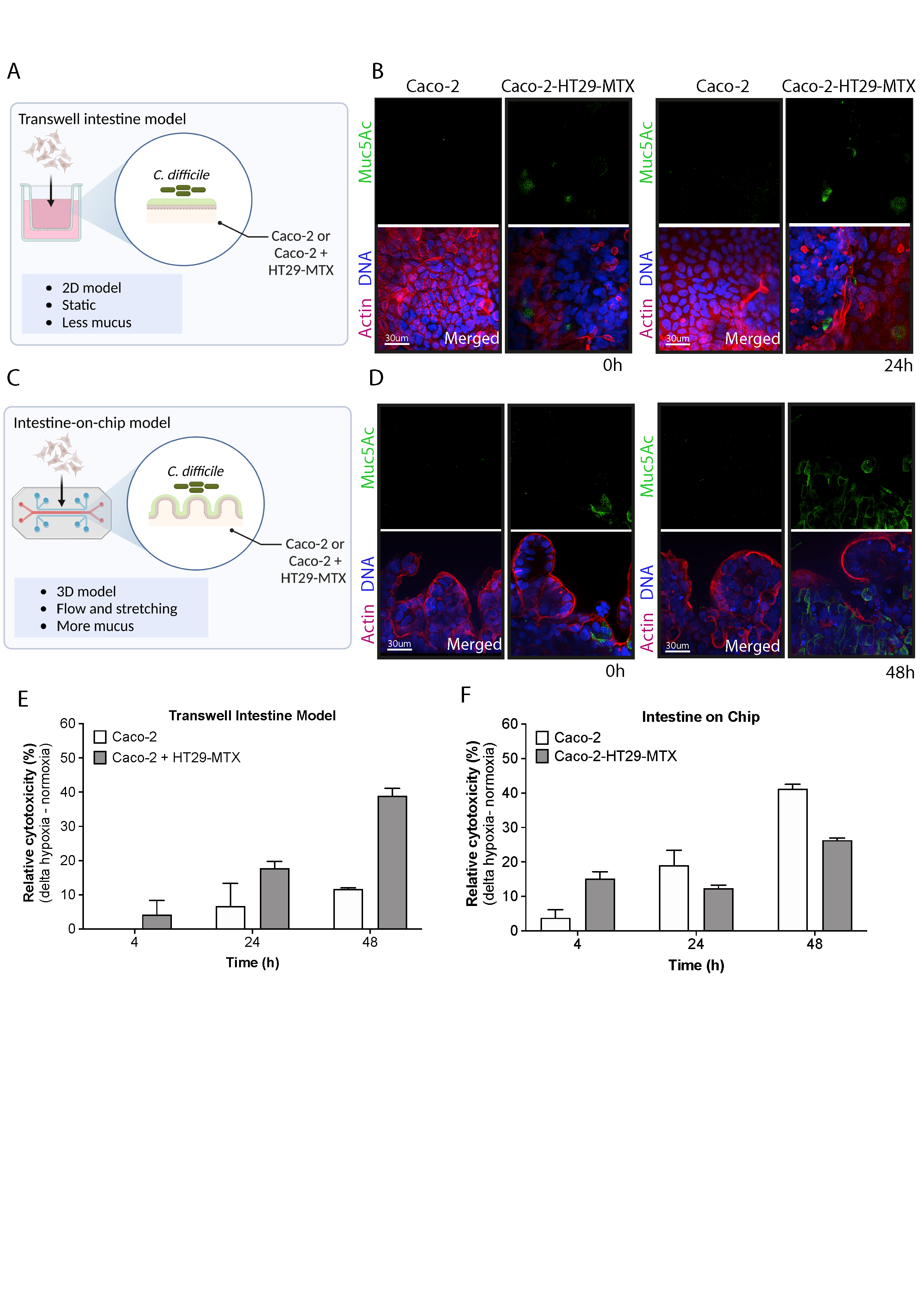


**Figure S1. Establishment of hypoxic intestinal models to study the role of the CDT binary toxin during *C. difficile* infection.** (A) Schematic representation of a Transwell Intestine Model (TIM) composed of Caco-2 cells alone or with HT29-MTX cells under hypoxia conditions (4% O_2_, 5% CO_2_). (B) Representative 3D reconstructed images of uninfected TIM under normoxia conditions (T0h, 5% CO_2_) and under hypoxia conditions (T24h, 4% O_2_, 5% CO_2_) (C). Schematic representation of the Intestine-on-chip model (IoC) composed of Caco-2 cells alone or with HT29-MTX cells under hypoxia conditions (4% O_2_, 5% CO_2_). (D) Representative 3D reconstructed images of uninfected IoC under normoxia conditions (T0h, 5% CO_2_) and under hypoxia conditions (T48h, 4% O_2_, 5 %CO_2_). DNA was labelled with DAPI (blue), mucin with anti-Muc-5AC AF488 (green) and actin with phalloidin rhodamine (red). Lactate dehydrogenase release assays were performed to evaluate cell survival in (E) the Transwell intestine model (TIM) and (F) the Intestine-on-chip (IoC) model after 4, 24 and 48h under normoxia or hypoxia (4% O_2_, 5% CO_2_). The relative cytotoxicity is represented as the difference between the hypoxia and normoxia conditions (delta hypoxia-normoxia). Delta values ≤40% were accepted. Data represents mean with SEM.


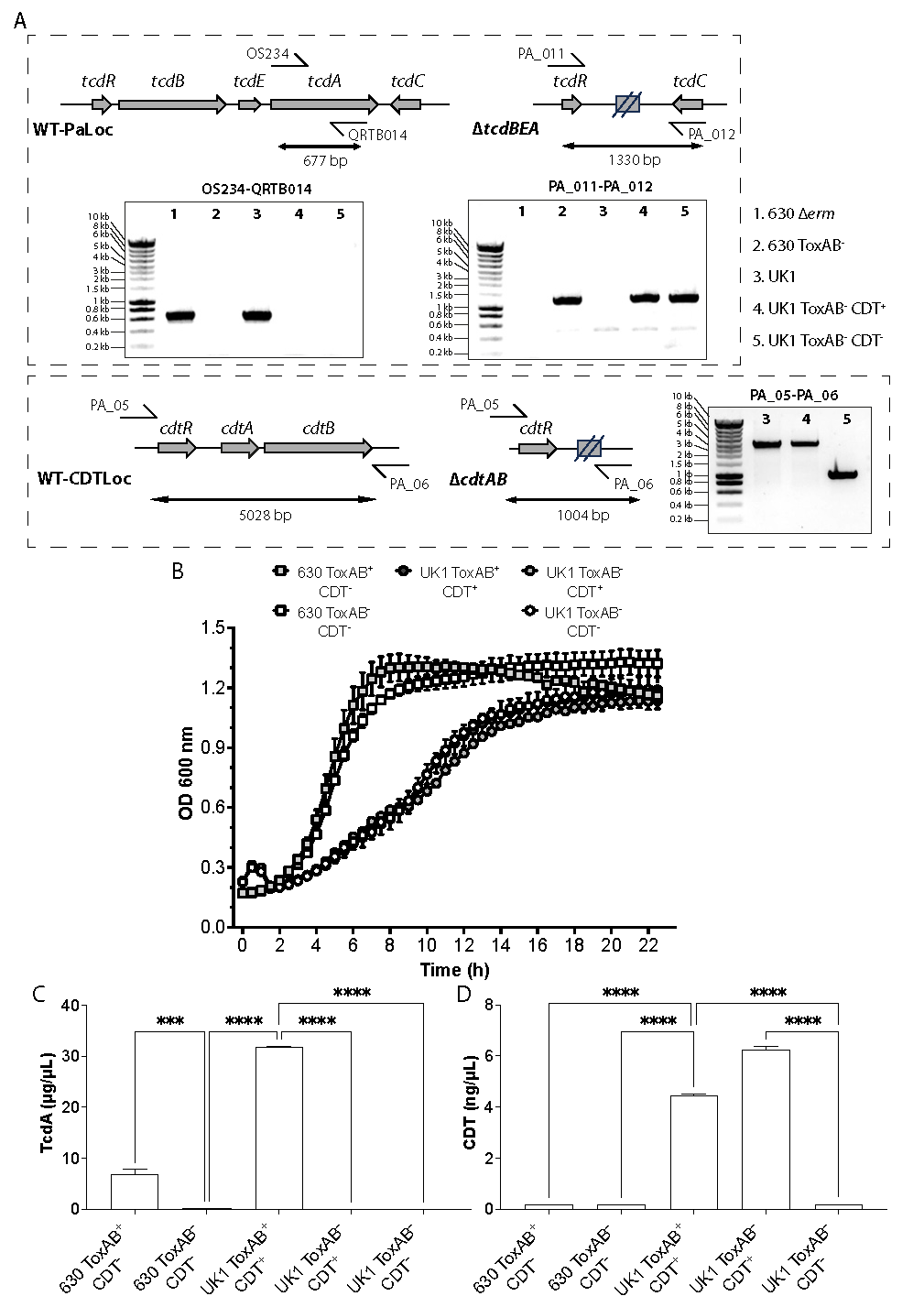


**Figure S2. Growth of generated PaLoc and CdtLoc *C. difficile* mutants and absence of TcdA and CDT toxins in culture supernatants A.** (A) PCR verification of the *tcdBEA* deletion mutants in both 630 Δ*erm* and UK1 strains and PCR verification of the *cdtAB* deletion mutants in derivative *tcdBEA* mutant of *C. difficile* UK1 strain. The pair OS234-QRTB014 that amplify an internal fragment of *tcdA* gene was used to verify the intact PaLoc, PA_011-PA_012 to verify the *tcdBEA* deletion and PA_05-PA_06 to verify the *cdtAB* deletion. (B) Growth curves of 630 Δ*erm*, UK1 WT or *tcdBEA* and *cdtAB* mutant strains in TY medium supplemented with glucose. (C) TcdA toxin or (D) CDT toxin secretion in extracellular fractions of 630 Δ*erm*, UK1 WT or *tcdBEA* and *cdtAB* mutant strains after 24h of growth in TY medium. A one-way ANOVA was performed and statistical significance is represented (***p <0.001 and **** p<0.0001). Data represents mean with SEM (C, D, E).

**
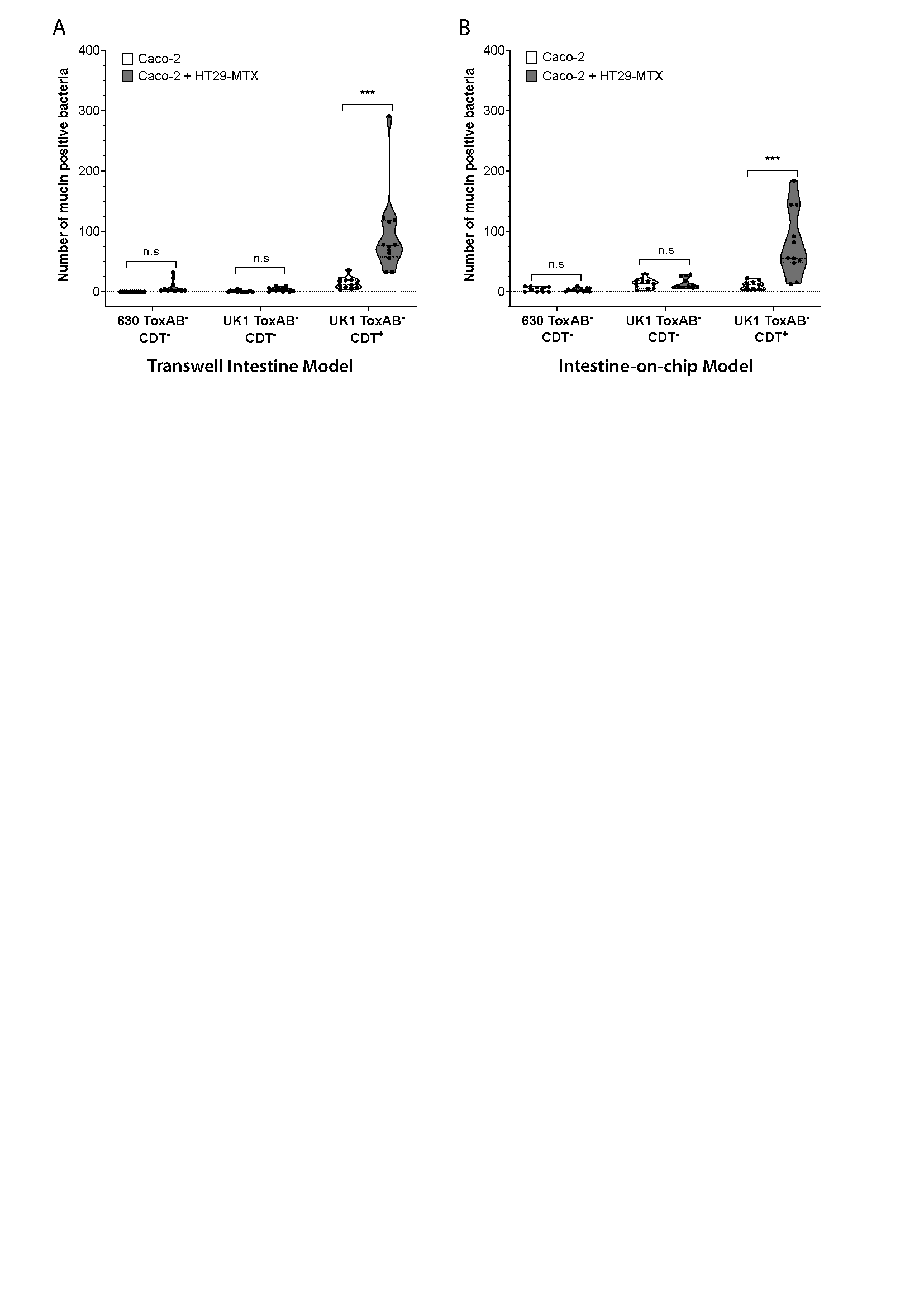
**

**Figure S3.** **CDT-dependent microcolonies co-localize with mucin in a Transwell Intestine model and Intestine-on-chip models.** Caco-2 alone or cocultured with HT29-MTX cells were infected with 630 ToxAB^-^CDT^-^, UK1 ToxAB^-^CDT^-^, or UK1 ToxAB^-^CDT^+^ under hypoxic conditions (4% O_2_, 5% CO_2_). Total mucin positive bacteria counted in Caco-2 cells alone or with HT29-MTX cells infected in (A) the TIM at 24h p.i or (B) the IoC model at 48h p.i with *C. difficile* strains as indicated. The number of mucin positive bacteria are reported for each image and at least 10 images were quantified. Each black square in the graph represents one image. Data and quantifications are representative of 2 (IoC) or 3 (TIM) independent biological replicates. Multiple unpaired *t* tests were performed and statistical significance is represented with ***p <0.001.

**
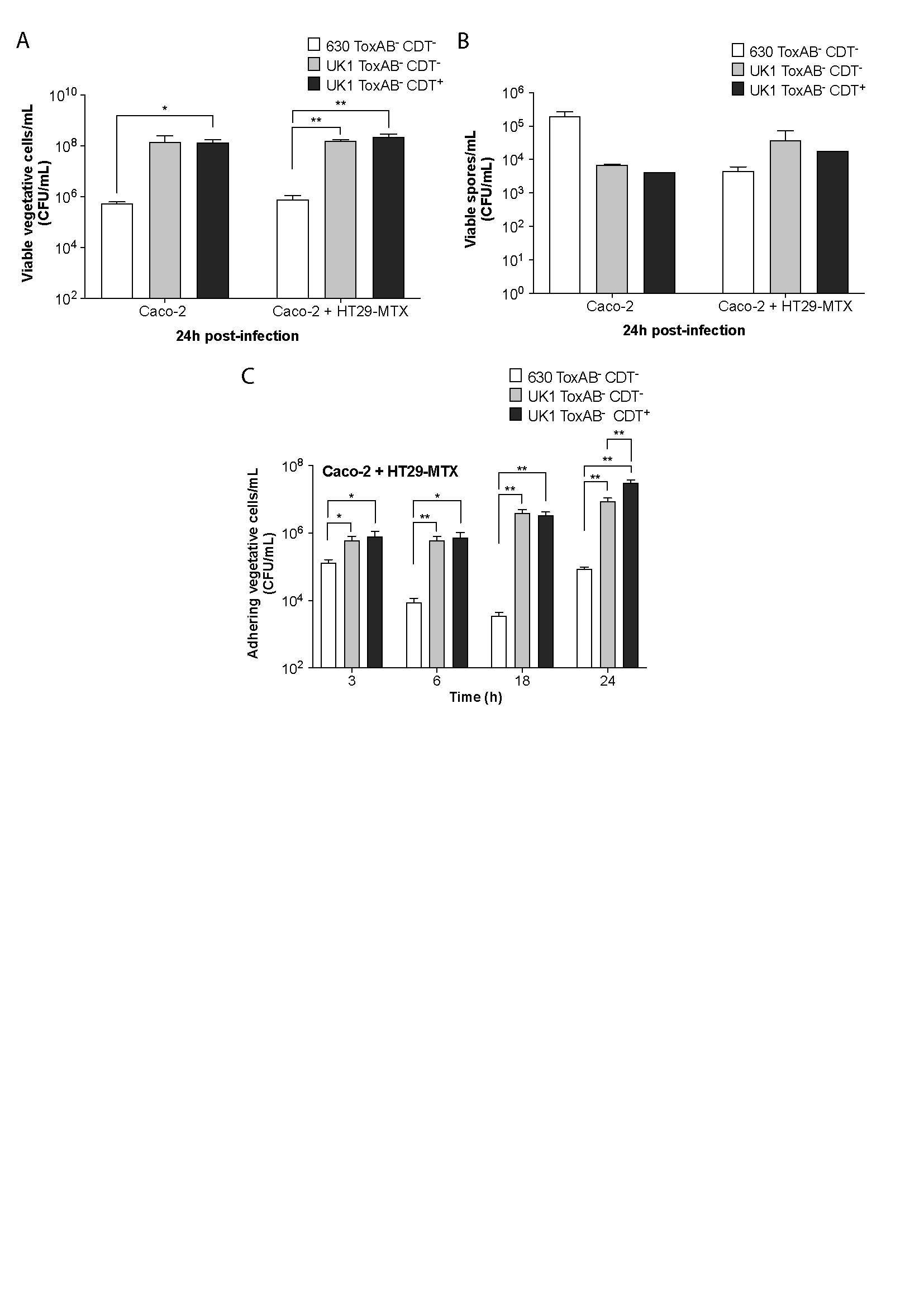
**

**Figure S4. Growth and adhesion of *C. difficile* strains in the Transwell Intestine model 24h after infection.** Caco-2 cells alone or co-cultured with HT29-MTX cells in the TIM model were infected with 630 ToxAB^-^CDT^-^, UK1 ToxAB^-^CDT^-^ and UK1 ToxAB^-^CDT^+^ strains. (A) Total viable vegetative cells (CFU) were numbered 24h p.i. (B) Adhering vegetative cells were numbered 24h p.i after eliminating non-adhering vegetative cells by PBS washes. Data represents mean with SD (A, C) or SEM (B). Multiple unpaired *t* tests were performed and statistical significance is represented (* p<0.05, ** p <0.01).

**
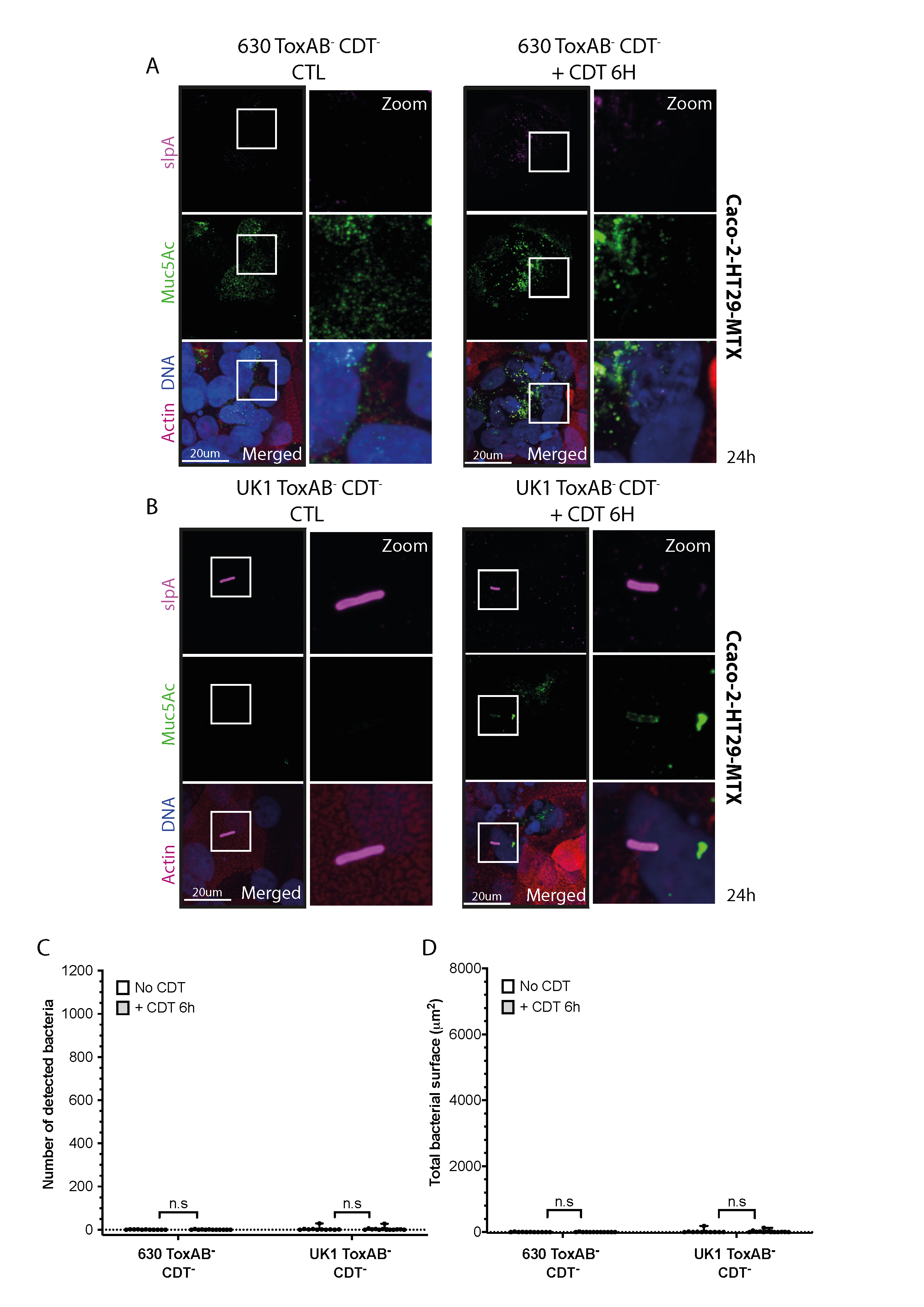
**

**Figure S5. CDT^-^ strains treated with purified CDT toxin during 6h in in the Transwell intestine model.** Representative 3D reconstructed images of Caco-2 cocultured with HT29-MTX cells infected with (A) 630 ToxAB^-^CDT^-^ or (B) UK1 ToxAB^-^CDT^-^ during 24h under hypoxic conditions (4% O_2_, 5% CO_2_). Infected intestinal cells were exposed to CdtA (200ng/mL) and activated CdtB (400 ng/mL) during 6h. DNA was labelled with DAPI (blue), mucin with anti-Muc5AC AF488 (green), actin with phalloidin rhodamine (red) and *C. difficile* with anti-SlpA AF647 (magenta). (C) Number of bacteria detected 24h p.i in Caco-2 cells cocultured with HT29-MTX cells infected with *C. difficile* strains as indicated. (D) Total bacteria surface detected 24h p.i in cells infected with *C. difficile* strains as indicated. The number of bacteria and total bacterial surface detected are reported for each image and at least 10 images were quantified per condition. Each black circle in the graph represents one image.

**
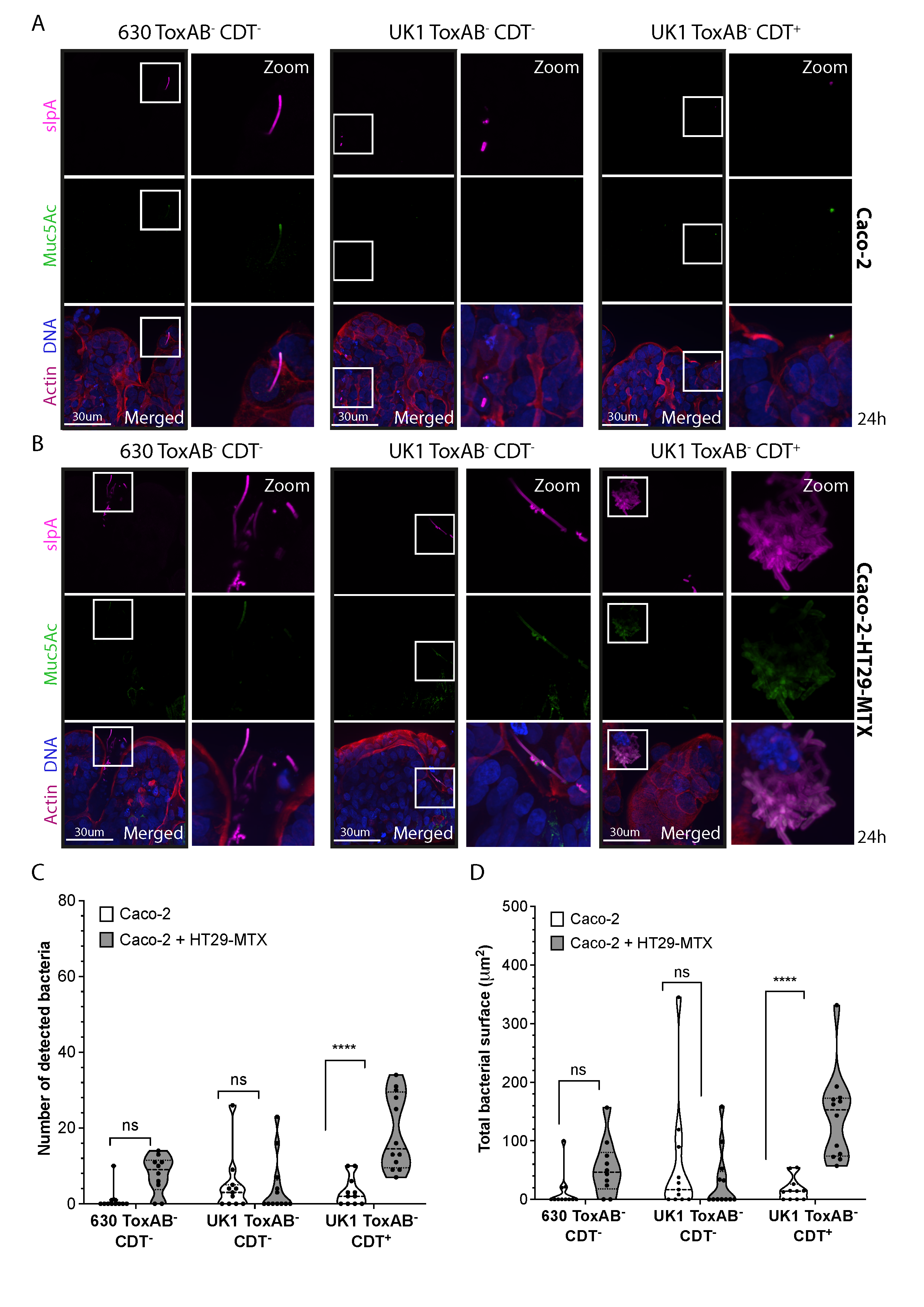
**

**Figure S6. CDT^+^ strain forms clumps in an Intestine-on-chip model 24h post-infection.** Representative 3D reconstructed images of (A) Caco-2 cells or (B) Caco-2 cells cocultured with HT29-MTX cells infected with 630 ToxAB^-^CDT^-^, UK1 ToxAB^-^CDT^-^ or UK1 ToxAB^-^CDT^+^ during 24h under hypoxic conditions as previously indicated. DNA was labelled with DAPI (blue), anti-Muc5AC AF488 (green), actin with phalloidin rhodamine (red) and *C. difficile* with anti-SlpA AF647 (magenta). (C) Number of bacteria detected 24h p.i in Caco-2 cells alone or with HT29-MTX cells infected with *C. difficile* strains as indicated. (C) Total bacteria surface detected 24h p.i in Caco-2 cells alone or with HT29-MTX cells infected with *C. difficile* strains as indicated. The number of bacteria and total bacterial surface detected are reported for each image and at least 10 images were quantified per condition. Each black circle in the graph represents one image. Data and quantifications are representative of 1 independent biological replicate. Multiple unpaired *t* tests were performed and statistical significance is represented with **** p<0.0001.


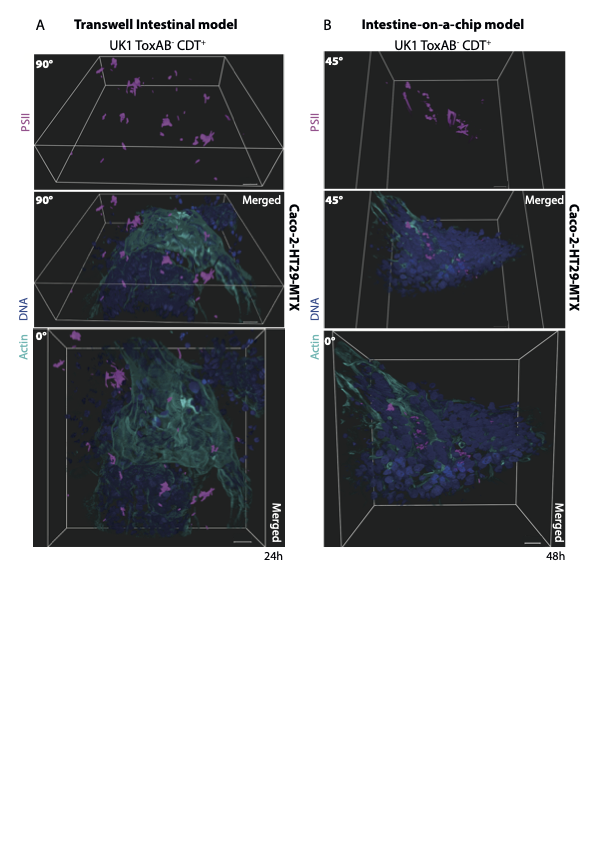


**Figure S7. CDT mucin-associated microcolonies do not show fiber-like polysaccharides structures.** Representative 3D images reconstructed with blend mode. Caco-2 cells cocultured with HT29-MTX cells in the TIM (A) or IoC model (B) were infected with UK1 ToxAB^-^CDT^+^ during 24h or 48h as indicated under hypoxic conditions (4%O_2_, 5%CO_2_). DNA was labelled with DAPI (blue), actin with phalloidin rhodamine (cyan) and *C. difficile* with anti- anti-PSII antibody AF647 (magenta).

**
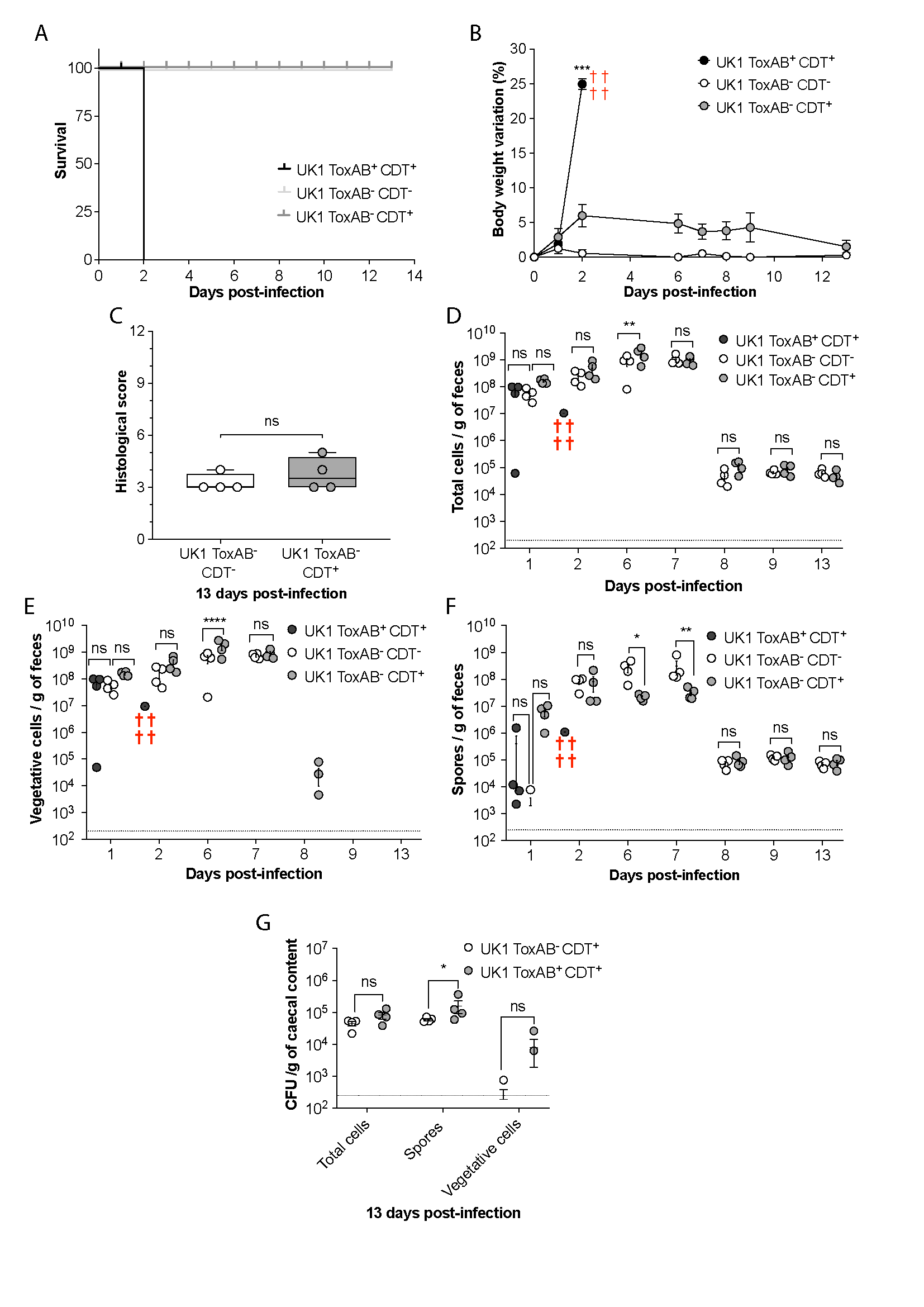
**

**Figure S8. Mice survival, body weight and *C. difficile* shedding into feces or caecal content.** C57Bl/6J Germ free mice 7-week old were infected with UK1 ToxAB^+^CDT^+^, UK1 ToxAB^-^CDT^-^ or UK1 ToxAB^-^CDT^+^. (A) Mice survival after infection with *C. difficile* strains as indicated. (B) Body weight average variation (%) of mice infected with *C. difficile* strains as indicated. (C) Histological score 13 days p.i. (D) Total cells (spores + vegetative cells) were analyzed different days p.i. (E) Spores were analyzed different days p.i. (F) Vegetative cells were analyzed different days p.i. (G) Total cells, spores and vegetative cells detected from caecal content from mice infected with *C. difficile* strains as indicated 13 days p.i. Data represents mean with SEM. Multiple unpaired *t* tests were performed and statistical significance is represented with * p<0.05, ** p <0.01, ***p <0.001 and, **** p<0.0001. ns: no statistical significance.

**SUPPLEMENTAL TABLES**

**Table S1. Strains and plasmids used in this study.**

| **Strain** | **Genotype** | **Origin** |
| --- | --- | --- |
| ***E. coli*** |  |  |
| NEB-10 beta | Δ*(ara-leu) 7697 araD139  fhuA* Δ*lacX74 galK16 galE15 e14-* ϕ*80*d*lacZ*Δ*M15  recA1 relA1 endA1 nupG  rpsL* (Str^R^) *rph spoT1* Δ*(mrr-hsdRMS-mcrBC)* | New England Biolabs |
| HB101(RP4) | *supE44* *aa14 galK2 lacY1* Δ(*gpt-proA*) 62 *rpsL20 (*Str^R^*)xyl-5* *mtl-1 recA13* Δ(*mcrC-mrr*) *hsdS*_B_ (r_B_-m_B_-) RP4 (Tra^+^ IncP Ap^R^ Km^R^ Tc^R^) | Laboratory stock |
| ***C. difficile*** |  |  |
| 630*∆erm* | 630*∆erm* 012 | Reference^1^ |
| CD1611 | *630∆erm* ToxAB^-^ | This study |
| CD1666 | UK1 | Reference^2^ |
| CNRS_CD129 | UK1 ToxAB^-^ CDT^+^ | This study |
| CNRS_CD345 | UK1 ToxAB^-^ CDT^-^ | This study |
| **Plasmid** |  |  |
| pMSR0 | Allele exchange for C*. difficile* UK1 | Reference^3^ |
| p127 | pMSR derivative for CdtLoc deletion | This study |
| p128 | pMSR derivative for PaLoc deletion | This study |

**Table S2. Oligonucleotides used in this study.**

| **Primer** | **Sequence (5’ to 3’)*** | **Use** |
| --- | --- | --- |
| PA_001 | ttttttgttaccctaagtttCATGATATGGAAATTGCTG | 5’ left arm for *cdtAB* deletion |
| PA_002 | gagtaattgcCATTTATTCTCCCTCCCAATATTAG | 3’ left arm for *cdtAB* deletion |
| PA_003 | agaataaatgGCAATTACTCCAGACGATAG | 5’ right arm for *cdtAB* deletion |
| PA_004 | agattatcaaaaaggagtttGCAGAAAAAGCCGAAAAAC | 3’ right arm for *cdtAB* deletion |
| PA_005 | GTGATGGATTATGGATAGC | 5’ *cdtAB* deletion screening |
| PA_006 | TGTGGGGACAAATTTAAATC | 3’ *cdtAB* deletion screening |
| PA_007 | ttttttgttaccctaagtttGTTTGTTTTAGCAAGAAATAACTCAG | 5’ left arm for *tcdBEA* deletion |
| PA_008 | tattttagccCATAAAATTTTCTCCTTTACTATAATATTTTTATTG | 3’ left arm for *tcdBEA* deletion |
| PA_009 | aaattttatgGGCTAAAATATATGTTTGACAAAAAATTATTC | 5’ right arm for *tcdBEA* deletion |
| PA_010 | agattatcaaaaaggagtttCTTGTTCTGAAGACCATG | 3’ right arm for *tcdBEA* deletion |
| PA_011 | GAGAGGATGATTTTATGC | 5’ *tcdBEA* deletion screening |
| PA_012 | CCATACCAGGGATAGCTGTAG | 3’ *tcdBEA* deletion screening |
| QRTBD014 | ATAAATTGCATGTTGCTTCATAACT | Intact PaLoc in wild type strains |
| OS234 | AGCTTTCGCTTTAGGCAGTG | Intact PaLoc in wild type strains |
| Muc5AC Fw | TGATCATCCAGCAGCAGGGCT | qPCR |
| Muc5AC Rv | CCGAGCTCAGAGGACATATGGG | qPCR |
| Muc1c Fw | ACTACTACCAAGAGCTG | qPCR |
| Muc1c Rv | CTCATAGGATGGTAGGT | qPCR |
| Muc2 Fw | ACTGCACATTCTTCAGCTGC | qPCR |
| Muc2 Rv | ATTCATGAGGACGGTCTTGG | qPCR |
| Rps13 Fw | CGAAAGCATCTTGAGAGGAACA | qPCR |
| Rps13 Rv | TCGAGCCAAACGGTGAATC | qPCR |

*Lowercase bases indicate overlapping sequences

**Table S3. Susceptibility of *C. difficile* strains to vancomycin and fidaxomicin cultured in supplemented ADMEM medium**

| **Strains** | **Antibiotics** | **Reported MIC for WT (μg/mL)** | **MIC ADMEM (μg/mL)** | **Reference** |
| --- | --- | --- | --- | --- |
| UK1 WT | Vancomycin | 1 | 12.5 | ^4^ |
| UK1 ToxAB^-^ CDT^+^ |  |  |  |  |
| UK1 ToxAB^-^ CDT^-^ |  |  |  |  |
| UK1 WT | Fidaxomicin | 0.25 | 1 | ^5^ |
| UK1 ToxAB^-^ CDT^+^ |  |  |  |  |
| UK1 ToxAB^-^ CDT^-^ |  |  |  |  |

**References**

1. Hussain HA, Roberts AP, Mullany P. Generation of an erythromycin-sensitive derivative of Clostridium difficile strain 630 (630Δerm) and demonstration that the conjugative transposon Tn916ΔE enters the genome of this strain at multiple sites. Journal of Medical Microbiology 2005; 54:137–41.

2. Killgore G, Thompson A, Johnson S, Brazier J, Kuijper E, Pepin J, Frost EH, Savelkoul P, Nicholson B, Van Den Berg RJ, et al. Comparison of Seven Techniques for Typing International Epidemic Strains of *Clostridium difficile* : Restriction Endonuclease Analysis, Pulsed-Field Gel Electrophoresis, PCR-Ribotyping, Multilocus Sequence Typing, Multilocus Variable-Number Tandem-Repeat Analysis, Amplified Fragment Length Polymorphism, and Surface Layer Protein A Gene Sequence Typing. J Clin Microbiol 2008; 46:431–7.

3. Peltier J, Hamiot A, Garneau JR, Boudry P, Maikova A, Hajnsdorf E, Fortier L-C, Dupuy B, Soutourina O. Type I toxin-antitoxin systems contribute to the maintenance of mobile genetic elements in Clostridioides difficile. Commun Biol 2020; 3:718.

4. Garneau JR, Valiquette L, Fortier L-C. Prevention of Clostridium difficile spore formation by sub-inhibitory concentrations of tigecycline and piperacillin/tazobactam. BMC Infect Dis 2014; 14:29.

5. Goldstein EJC, Citron DM, Sears P, Babakhani F, Sambol SP, Gerding DN. Comparative Susceptibilities to Fidaxomicin (OPT-80) of Isolates Collected at Baseline, Recurrence, and Failure from Patients in Two Phase III Trials of Fidaxomicin against Clostridium difficile Infection. Antimicrob Agents Chemother 2011; 55:5194–9.
